# Rab35 GTPase couples cell division with initiation of epithelial apico-basal polarity and lumen opening

**DOI:** 10.1038/ncomms11166

**Published:** 2016-04-04

**Authors:** Kerstin Klinkert, Murielle Rocancourt, Anne Houdusse, Arnaud Echard

**Affiliations:** 1 Membrane Traffic and Cell Division Lab, Cell Biology and Infection Department, Institut Pasteur, 25–28 rue du Dr Roux, 75724 Paris, France; 2 Centre National de la Recherche Scientifique UMR3691, 75015 Paris, France; 3 Sorbonne Universités, Université Pierre et Marie Curie, Université Paris 06, Institut de formation doctorale, 75252 Paris, France; 4 Institut Curie, Structural Motility Lab, 26 rue d'Ulm, 75005 Paris, France

## Abstract

Establishment and maintenance of apico-basal polarity in epithelial organs must be tightly coupled with cell division, but the underlying molecular mechanisms are largely unknown. Using 3D cultures of renal MDCK cells (cysts), we found that the Rab35 GTPase plays a crucial role in polarity initiation and apical lumen positioning during the first cell division of cyst development. At the molecular level, Rab35 physically couples cytokinesis with the initiation of apico-basal polarity by tethering intracellular vesicles containing key apical determinants at the cleavage site. These vesicles transport aPKC, Cdc42, Crumbs3 and the lumen-promoting factor Podocalyxin, and are tethered through a direct interaction between Rab35 and the cytoplasmic tail of Podocalyxin. Consequently, Rab35 inactivation leads to complete inversion of apico-basal polarity in 3D cysts. This novel and unconventional mode of Rab-dependent vesicle targeting provides a simple mechanism for triggering both initiation of apico-basal polarity and lumen opening at the centre of cysts.

## Abstract (web summary)

Establishment and maintenance of apico-basal polarity in epithelial organs needs to be tightly coupled with cell division. Here the authors show that the Rab35 GTPase tethers intracellular vesicles containing key apical determinants at the cleavage site, connecting cytokinesis to apico-basal polarity.

## Graphical abstract

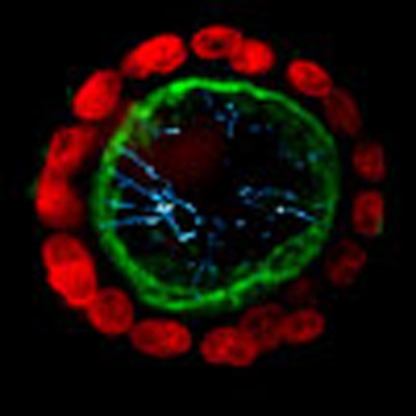

Many epithelial organs are composed of a polarized cell monolayer surrounding a central apical lumen. Renal Madin-Darby canine kidney (MDCK) cells cultured in Matrigel form polarized hollow spheres (cysts) that represent a powerful model for deciphering the establishment of epithelial polarity and lumen formation^1^,^2^,^3^,^4^,^5^. The *de novo* apico-basal polarity in cysts arises from successive divisions of a single, non-polarized cyst-founding cell^6^,^7^. During the first cell division, apical transmembrane proteins such as Podocalyxin (PODXL, a classical apical marker also known as GP135) and Crumbs3 are transcytosed from the plasma membrane facing the extracellular matrix (ECM) towards the first cell–cell contact site^8^,^9^,^10^. Membrane traffic is therefore essential for this symmetry-breaking step, which specifies the location of the apical membrane initiation site (AMIS) and thus the central position of the future apical lumen^10^,^11^,^12^. Recent data indicate that the location of the cytokinetic bridge between the first two daughter cells determines the location of the AMIS^12^,^13^,^14^,^15^, yet the molecular mechanisms coupling the first cell division with apical lumen formation are largely unknown. We previously identified Rab35 as a unique Rab GTPase present at the cleavage site that promotes cytokinetic abscission in HeLa cells^16^,^17^,^18^,^19^,^20^. Given the postulated analogy between the cytokinetic plasma membrane and the apical membrane of polarized epithelial cells^21^, we hypothesized that Rab35 might play a role in apico-basal polarity events. Here we show that the Rab35 GTPase is localized at the cell–cell interface and at the future AMIS during cytokinesis, where it captures vesicles transporting key apical determinants via a direct interaction with the cytoplasmic tail of PODXL. Through this original mechanism of vesicle tethering, Rab35 thus couples cell division with initiation of apico-basal polarity and lumen formation.

## Results

### Rab35 directly interacts with PODXL at the apical membrane

**Figure 1:**
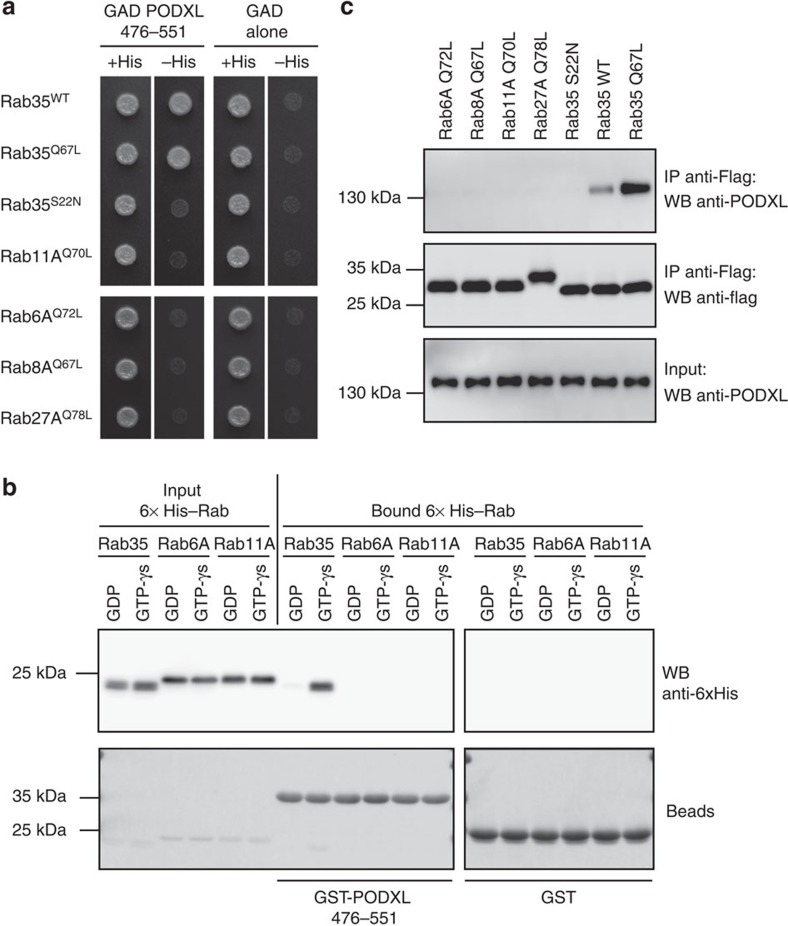
GTP-bound Rab35 directly interacts with PODXL. (**a**) *Saccharomyces cerevisiae* L40 reporter strain was transformed with plasmids encoding GAD fused to PODXL cytoplasmic tail (aa 476–551) or GAD alone to examine interactions with LexA fused to Rab35^WT^, Rab35^Q67L^ (GTP-bound mutant), Rab35^S22N^ (GDP-bound mutant), Rab11A^Q70L^ (GTP-bound mutant), Rab6A^Q72L^ (GTP-bound mutant), Rab8A^Q67L^ (GTP-bound mutant) and Rab27A^Q78L^ (GTP-bound mutant). Growth on a medium without histidine (−His) indicates an interaction with the corresponding proteins in this two-hybrid assay. (**b**) Recombinant GST or GST–PODXL (aa 476–551) immobilized on glutathione beads was incubated with recombinant 6xHis-tagged Rab35^WT^, Rab11A^WT^ or Rab6A^WT^, and loaded with either GDP or GTPγS. Top panel: Rab proteins directly bound to beads were detected by western blot with anti-6xHis antibodies. 5% of the Rab protein input are displayed in the first six lanes. Bottom panel: bead inputs in Ponceau staining. (**c**) Rab proteins were immunoprecipitated with anti-Flag antibodies (IP) from MDCK cells transfected with plasmids encoding 3xFlag-tagged Rab6A^Q72L^, Rab8A^Q67L^, Rab27A^Q78L^, Rab11^Q70L^, Rab35^S22N^, Rab35^WT^ or Rab35^Q67L^. Flag-proteins and co-immunoprecipitated endogenous PODXL were detected by western blot using anti-PODXL antibodies (top panel) and anti-Flag antibodies (middle panel). Corresponding inputs (5% of total lysates) are displayed in the bottom panel.

**Figure 2:**
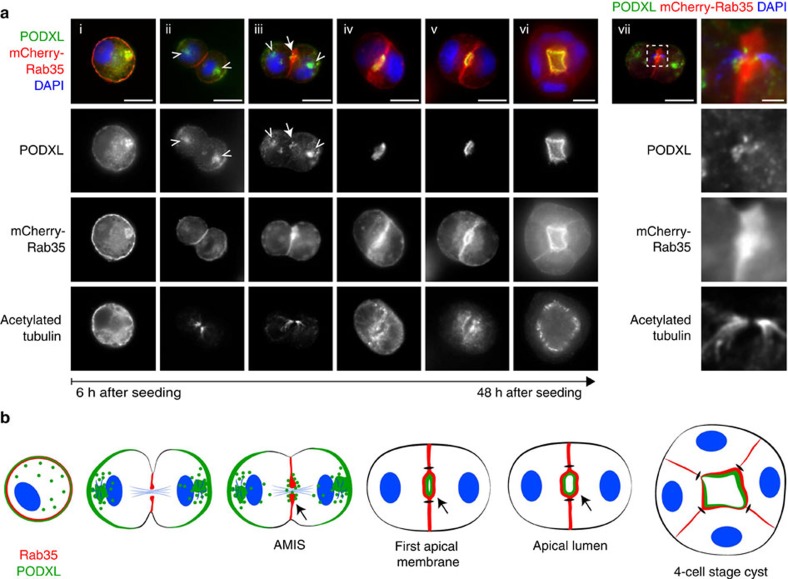
Rab35 and PODXL co-localize at the apical membrane since the first cytokinesis of 3D cyst development. (**a**) MDCK cells stably expressing mCherry-Rab35 were cultured in Matrigel and fixed between 6 and 48 h after seeding. Merged pictures show endogenous PODXL (green), DAPI (blue) and mCherry-Rab35 (red) in immunofluorescence. Corresponding channels for PODXL, mCherry-Rab35 and acetylated tubulin are displayed in grey levels. Arrowheads and arrows point towards endocytic recycling compartments and AMIS, respectively. vii is the same cyst as in iii, and the right panels correspond to the intercellular bridge region at higher magnification (merged images and single channels, as indicated). Scale bars, 10 μm (2 μm for zoomed region). (**b**) Localization summary of PODXL (green) and Rab35 (red) through cell division of the cyst-founding cell, two-cell cyst and four-cell cyst. Blue lines: intercellular bridge microtubules. Nuclei are figured in blue. Arrows point towards the AMIS and the first apical membrane.

We first identified a potential connection between Rab35 and PODXL through a yeast two-hybrid screen using the active, GTP-bound mutant of Rab35 (Rab35^Q67L^) as a bait. We found that the cytoplasmic tail of PODXL (aa 476–551) interacted selectively with Rab35^WT^ and Rab35^Q67L^, but not with Rab35^S22N^, the GDP-bound, inactive form (Fig. 1a). In contrast, no interaction was detected between the PODXL tail and the GTP-locked mutants of Rab6A or Rab GTPases involved in cystogenesis like Rab8, Rab11A or Rab27A (Fig. 1a). Using recombinant proteins, we confirmed that the PODXL/Rab35 interaction was direct and specific for the GTP-bound conformation of Rab35 (Fig. 1b). In addition, endogenous PODXL could be co-immunoprecipitated from MDCK cells expressing Rab35^WT^ or Rab35^Q67L^, but not from Rab35^S22N^-, Rab6A^Q72L^-, Rab8A^Q67L^-, Rab11^Q70L^- or Rab27A^Q78L^-expressing cells (Fig. 1c). To examine where this direct interaction takes place during cystogenesis, we stained for endogenous PODXL in MDCK cells stably expressing mCherry-Rab35. During initial phases of three-dimensional (3D) cyst development, PODXL vesicles concentrated on endosomal recycling compartments at the two-cell stage (arrowheads) and then concentrated at the AMIS (arrow), as previously reported^9^,^10^,^14^ (Fig. 2a(iii)). Importantly, we noticed that Rab35 was present at the first cleavage furrow before any detectable co-localization with PODXL (Fig. 2a(ii)). Early signs of co-localization were observed when PODXL started to be trafficked towards the cytokinetic bridge (Fig. 2a(iii), and zoom (vii)). A remarkable close apposition between PODXL-containing vesicles and membrane-bound Rab35 was thus initially detected at the AMIS. Subsequently, PODXL strongly co-localized with Rab35 at the AMIS and at the apical membrane (Fig. 2a(iv–vi) and Fig. 2b). We observed that Rab35 was not restricted to the AMIS (defined by ZO-1) and that part of Rab35 also localized on its sides (β-catenin positive) at the first cell–cell interface (Supplementary Fig. 1a,b and Discussion). Altogether, these results indicate that PODXL is a genuine Rab35-interacting protein and suggest that Rab35 could play a critical role in early steps of cyst development.

### Rab35 depletion leads to a complete inversion of polarity

**Figure 3:**
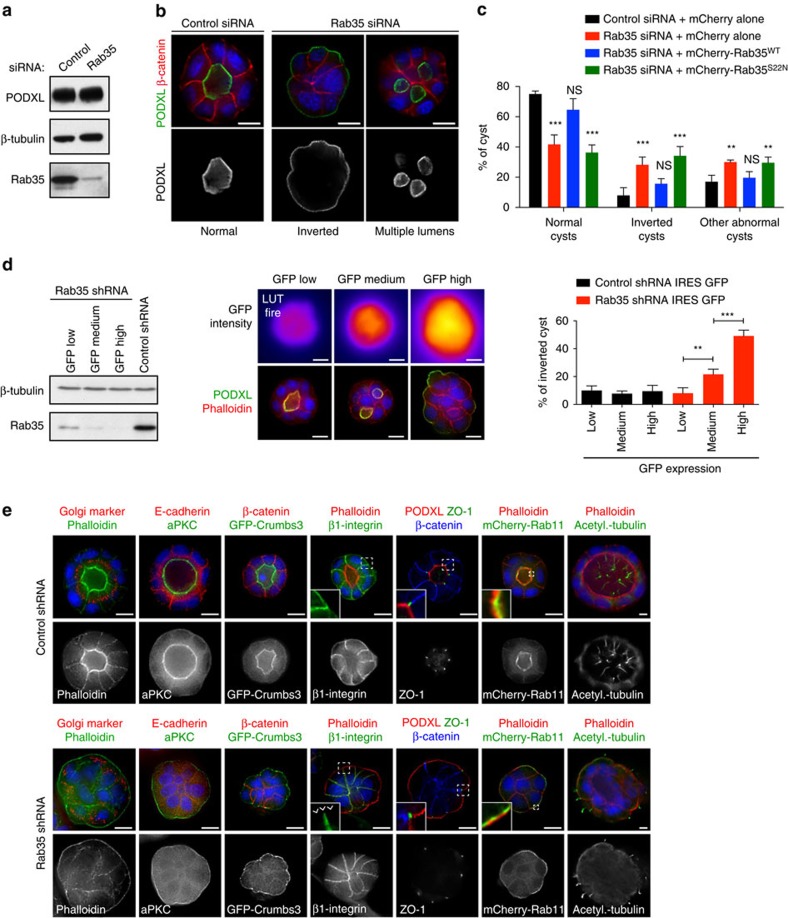
Rab35 depletion leads to a complete inversion of cyst apico-basal polarity. (**a**) Western blot of MDCK cell lysates after control or Rab35 depletion, using antibodies detecting PODXL, β-tubulin and Rab35, as indicated. (**b**) MDCK cells were treated with control or Rab35 siRNAs and seeded into Matrigel for 48 h. Cysts were fixed and stained for DAPI (blue), PODXL (green) and β-catenin (red) or PODXL as single channel (grey), as indicated. Examples of an inverted cyst or a multiple lumen cyst observed after Rab35 depletion are displayed. Scale bar, 10 μm. (**c**) MDCK cells were treated with control or Rab35 siRNAs, then transfected with plasmids encoding mCherry alone, siRNA-resistant mCherry-Rab35^WT^ or siRNA-resistant mCherry-Rab35^S22N^. Cysts were fixed after 48 h in Matrigel and categorized as normal cysts, inverted cysts or other abnormal cysts based on PODXL staining. Mean±s.d., *N*=3 independent experiments, 300–900 cysts analysed per condition. Two-way analysis of variance (ANOVA): **P<0.01; ***P<0.001; NS, not significant. (**d**) Left panels: lysates from Rab35 shRNA IRES GFP MDCK cells sorted according to low, medium and high levels of fluorescence were analysed by western blot, as indicated. Note that Rab35 levels are the most reduced in the highest GFP cells. Middle panels: cells with indicated intensity of GFP fluorescence (top row, LUT fire) were seeded into Matrigel for 48 h, fixed and stained for PODXL (green), F-actin by phalloidin (red) and DAPI (blue). Right panels: quantification of the proportion of inverted cysts based on PODXL localization in MDCK cells expressing either control shRNA (black) or Rab35 shRNA (red), and indicated categories of GFP intensity. Mean±s.d., *N*=3 independent experiments, 300–1,500 cysts analysed per condition. Two-way ANOVA: **P<0.01; ***P<0.001; NS, not significant. (**e**) Control- or Rab35-depleted MDCK cells were cultured in Matrigel for 48 h (except for acetylated (acetyl.) tubulin: 7 days), fixed and stained for apical components (PODXL, F-actin (phalloidin), primary cilia (acetylated tubulin), aPKC, GFP-Crumbs3 and mCherry-Rab11 (sub-apical)), baso-lateral markers (β-catenin, E-cadherin and β1-integrin) or Golgi apparatus (CTR433 marker), as indicated. Selected channels are also displayed in grey levels. Scale bar, 10 μm.

To test the potential function of Rab35 in apico-basal polarity establishment and PODXL-dependent lumen formation, we depleted Rab35 using independent siRNAs (Fig. 3a and Supplementary Fig. 2a) or shRNAs (Supplementary Fig. 2b,c), and seeded single MDCK-depleted cells into Matrigel for 48 h. In control siRNA conditions, the majority of cysts (>75%) developed a single, PODXL-positive central lumen surrounded by an epithelial monolayer (Fig. 3b,c). In contrast, only 40% of Rab35-depleted cysts formed a single apical lumen. Remarkably, Rab35 depletion led to the appearance of cysts with inverted apico-basal polarity, characterized by mislocalization of PODXL to the outer, ECM-facing plasma membrane (Fig. 3b,c). The remaining Rab35-depleted cysts displayed either multiple lumens or intracellular vacuoles (‘other abnormal cysts'; Fig. 3b,c and Supplementary Fig. 2a). All these phenotypes were fully rescued with siRNA-resistant Rab35^WT^ but not with the Rab35^S22N^ mutant (Fig. 3c). The same defects were confirmed using an shRNA targeting a different region of *Rab35* mRNA, and were similarly rescued with Rab35^WT^ but not with the GDP-bound Rab35 (Supplementary Fig. 2b,c). In addition, expression of the Rab35 GAP EPI64B/TBC1D10B (refs 22, 23, 24) phenocopied Rab35 depletion (Supplementary Fig. 2d,e). Thus, both depletion and inactivation of Rab35 lead to defects in cyst development.

Using a Rab35 shRNA-IRES-GFP MDCK cell line, we established that fluorescence-activated cell sorting (FACS)-sorted cells that expressed the highest levels of green fluorescent protein (GFP) corresponded to the most Rab35-depleted cells (Fig. 3d). Interestingly, the most-depleted cells were the most prone to develop into inverted cysts (up to 50% of cysts, Fig. 3d, and full rescue by Rab35^WT^ in Supplementary Fig. 2f), indicating that polarity inversion is observed when Rab35 is reduced below a critical threshold. We thus focused on understanding why Rab35 depletion led to this striking phenotype. We first investigated whether polarity markers other than PODXL were also inverted upon Rab35 depletion. Whereas the classical apical markers F-actin, aPKC and Crumbs3 were strongly enriched at the central apical membrane in control cysts, they were all delocalized to the peripheral membrane facing the ECM after Rab35 depletion (Fig. 3e). Consistent with genuine inversion of polarity, the baso-lateral marker β1-integrin was absent from the membrane facing the ECM (Fig. 3e). Of note, lateral adherens junctions stained by E-cadherin formed correctly (Fig. 3e). In addition, ZO-1-labelled tight junctions were present, but with inverted location (Fig. 3e). Intracellular organization was also inverted, since sub-apical compartments such as the Golgi apparatus or the recycling compartment labelled by Rab11 were delocalized underneath the membrane facing the ECM (Fig. 3e). Finally, primary cilia, which always form at the apical luminal membrane in control cysts, sprouted from the ECM-facing membrane after Rab35 depletion (Fig. 3e). Altogether, we conclude that strong Rab35 depletion leads to a complete inversion of apico-basal polarity in cysts.

### Ectopic fusion of PODXL vesicles upon Rab35 depletion

**Figure 4:**
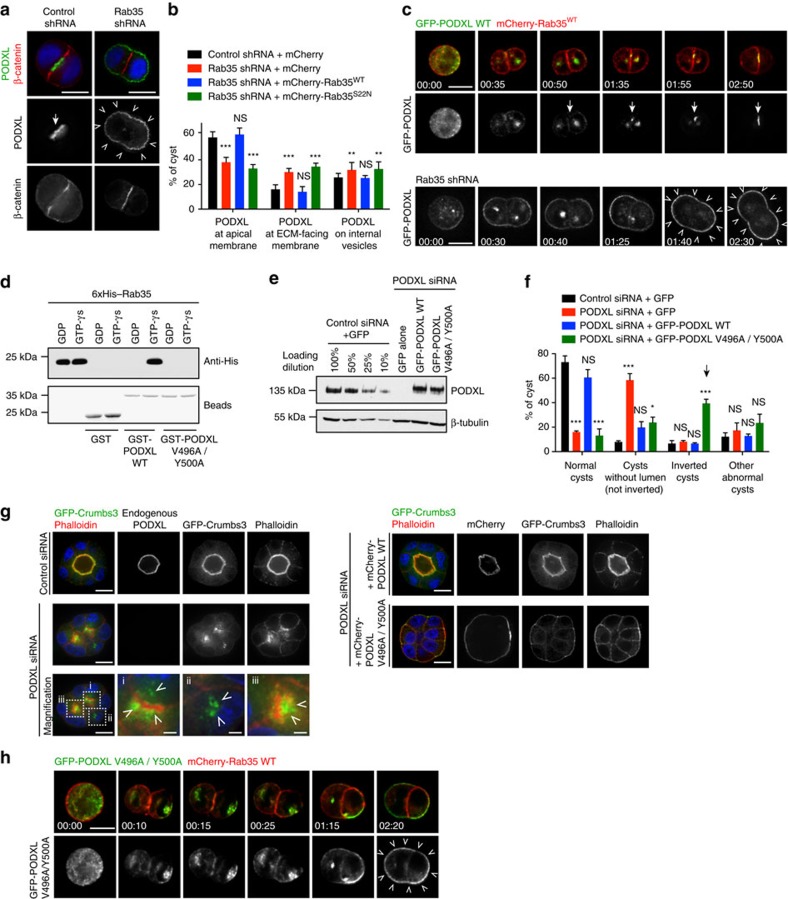
Ectopic fusion of PODXL-positive vesicles to the ECM-facing membrane on Rab35 depletion or disruption of Rab35/PODXL interaction. (**a**) Staining for PODXL, β-catenin and DAPI in control- or Rab35-depleted two-cell cysts fixed 24 h after seeding in Matrigel (merge and single channels, as indicated). Scale bar, 10 μm. (**b**) Localization of PODXL at the two-cell-stage cyst (24 h in Matrigel) after indicated depletion and plasmid transfection. Mean±s.d., *N*=3 independent experiments, 150–200 two-cell cysts analysed per condition. Two-way analysis of variance (ANOVA): **P<0.01, ***P<0.001, NS, not significant. (**c**) Top two rows: snapshots of time-lapse microscopy from control MDCK cells seeded into Matrigel and expressing mCherry-Rab35^WT^ (red) together with GFP-PODXL (green). Bottom row: snapshots of time-lapse microscopy from Rab35 shRNA MDCK cells seeded into Matrigel and expressing GFP-PODXL. Arrowheads indicate ectopic localization of PODXL after the first division. Scale bar, 10 μm. Time stamps: (hour:min) using mitotic entry as origin. (**d**) GST-pull down experiment as described in Fig. 1b, using either GST alone, GST–PODXL tail (aa 476–551) as in WT or GST–PODXL tail (aa 476–551) with the two substitutions V496A and Y500A. (**e**) MDCK cells treated with PODXL siRNAs for 3 days and transfected with indicated plasmids. Western blot showing PODXL (loading control: β-tubulin). For comparison, dilutions of lysates from control siRNA-treated MDCK cells transfected with plasmids encoding GFP alone have been used. 100% correspond to the amount loaded in the PODXL siRNA conditions. (**f**) Proportion of normal cysts, cysts without lumen (but not inverted), inverted cysts and other abnormal cysts following seeding of the cells described in **e**. in Matrigel for 48 h. Mean±s.d., *N*=3 independent experiments, 150–500 cysts analysed per condition. Two-way ANOVA: *P<0.05; ***P<0.001; NS, not significant. (**g**) MDCK cells as described in **e** were co-transfected with GFP-Crumbs3, fixed after 48 h in Matrigel and stained as indicated. Magnification: GFP-Crumbs3-positive vesicles close to the plasma membrane in displayed PODXL-depleted cyst. Scale bar, 10 μm (2 μm for zoomed region). (**h**) Snapshots of time-lapse microscopy from PODXL-depleted MDCK cells seeded into Matrigel and expressing mCherry-Rab35^WT^ (red) together with siRNA-resistant GFP-PODXL full-length V496A Y500A mutant (green). Single PODXL channel also displayed in grey levels. Arrowheads indicate ectopic localization of PODXL mutant after the first division. Scale bar, 10 μm.

We next addressed whether inversion of polarity upon Rab35 depletion occurred already in the initial steps of cyst development. As a first approach, we fixed control- and Rab35-depleted cysts 24 h after seeding single cells into Matrigel, and analysed PODXL localization at the two-cell stage (Fig. 4a). While 58% of control cysts displayed PODXL at the AMIS (Fig. 4a, arrow), this was the case in 38% of Rab35-depleted cysts (Fig. 4a,b). Interestingly, in line with the increase of inverted cysts observed at 48 h (Fig. 3c), Rab35 depletion led to an increase of cysts with PODXL abnormally located at the membrane facing the ECM already at 24 h (Fig. 4a, arrowheads). This abnormal localization was fully rescued by the expression of siRNA-resistant Rab35^WT^, but not of GDP-locked Rab35^S22N^ (Fig. 4b). We next turned to time-lapse microscopy of cells expressing GFP-PODXL to precisely characterize how inversion of polarity arose in Rab35-depleted cysts (Fig. 4c). In control cysts, PODXL was present at the plasma membrane and on vesicles during the first metaphase, then it was internalized into internal recycling compartments during telophase, but not yet localized at the AMIS (Supplementary Movie 1, Fig. 4c top panels, time 0:50; and also Fig. 2a(ii)). Finally, the recycling compartments from both cells moved towards the intercellular bridge and PODXL started to strongly accumulate at the AMIS (Fig. 4c, top panels, arrows). This first description in live cells of PODXL trafficking from the one-cell stage to the two-cell stage of cystogenesis is fully consistent with previous observations in fixed cysts, indicating that PODXL is transcytosed towards the AMIS at the two-cell stage^10^. Importantly, PODXL also reached the recycling compartments after Rab35 depletion (Fig. 4c, bottom panels, time 00:30–00:40; and Supplementary Movie 2). However, as these compartments moved towards the cell–cell interface, they became dimmer and a concomitant increase of PODXL back to the ECM-facing plasma membrane was observed. Two hours after mitosis onset, PODXL eventually localized abnormally to the ECM-facing membrane (Fig. 4c, bottom panels, arrowheads). Other examples of movie snapshots are presented in Supplementary Fig. 3. Taken together, these results indicate that inversion of polarity results from the inability of internalized PODXL-containing vesicles to fuse at the membrane surrounding the first cytokinetic bridge in Rab35-depleted cysts.

As Rab35 has been involved in the timing of cytokinetic abscission^17^, we investigated whether this could influence the establishment of polarity. We thus depleted Cep55, a protein critical for abscission in HeLa cells, and found indeed a strong delay in cytokinetic abscission in MDCK cells (Supplementary Fig. 4a). However, Cep55 depletion had no effect on apico-basal polarity in cysts (Supplementary Fig. 4b). Therefore, abscission delay cannot account for the inversion of polarity observed after Rab35 depletion.

To test whether the interaction between PODXL and Rab35 was essential for normal cyst polarity, we designed a C-terminal mutant of PODXL unable to bind Rab35 (Supplementary Fig. 4c). We reasoned that Rab GTPases usually interact with partners through hydrophobic amino acids, and screened for mutations that would disrupt the interaction. Using recombinant proteins, we found that the combination of the two point mutations V496A/Y500A in the PODXL cytoplasmic tail completely abolished the binding to Rab35 (Fig. 4d). We then replaced endogenous PODXL with either GFP alone, siRNA-resistant version of GFP-PODXL WT or GFP-PODXL V496A/Y500A (Fig. 4e). In PODXL-depleted cysts expressing GFP alone, we observed a striking increase of cysts without lumen but no polarity inversion (Fig. 4f). This was accompanied by the accumulation of apical recycling endosomes (positive for Crumbs3) close to the plasma membrane (Fig. 4g), as reported previously in ref. 25. This is consistent with a role of PODXL upstream or at the fusion step of these vesicles with the future apical membrane, as well as for lumen opening^25^,^26^,^27^. Ruling out off-target effects, the defects observed in PODXL-depleted cysts were fully rescued by expression of GFP-PODXL WT (Fig. 4f). Importantly, replacing endogenous PODXL with GFP-PODXL V496A/Y500A was sufficient to invert cyst polarity, without the need to deplete Rab35 (Fig. 4f, arrow). Time-lapse microscopy of the first cell division revealed that PODXL V496A/Y500A was internalized but failed to be correctly delivered to the presumptive AMIS, and instead accumulated back to the ECM-facing plasma membrane (Fig. 4h and Supplementary Movie 3). Thus, a PODXL mutant unable to interact with Rab35 triggers inversion of cyst polarity from the two-cell stage by preventing fusion of PODXL vesicles at the first cell–cell interface, exactly as observed after Rab35 depletion. Remarkably, fusion of the PODXL-positive vesicles to the ECM-facing membrane is sufficient to invert apical polarity, likely because they can transport key apical determinants such as aPKC, Cdc42 and Crumbs3 (refs 10, 14 and see below).

### GTP-bound Rab35 directly tethers PODXL-positive vesicles

**Figure 5:**
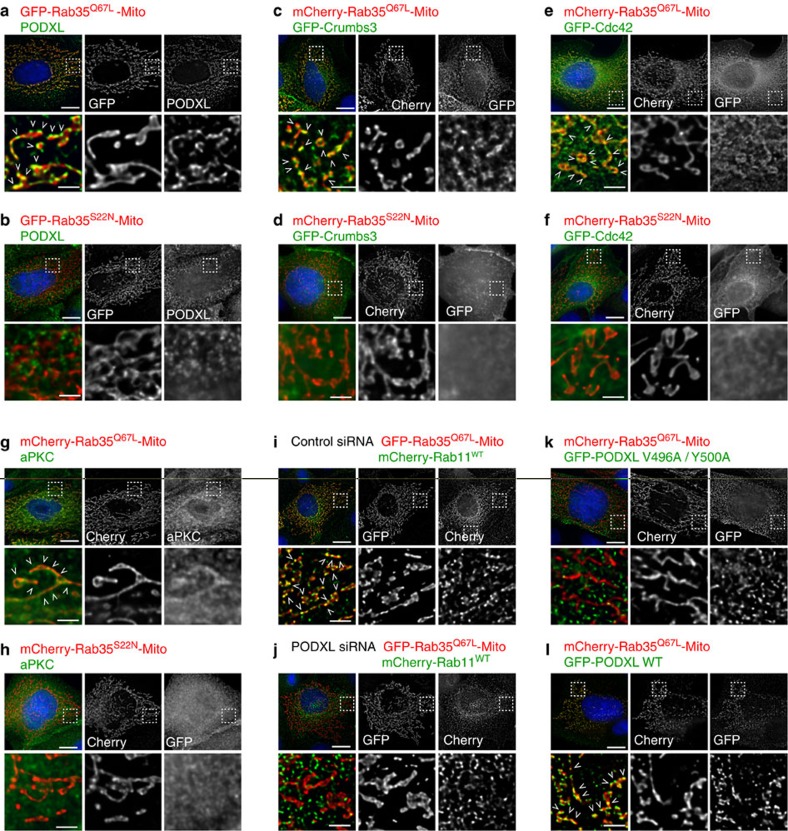
GTP-bound Rab35 acts a direct molecular tether for PODXL-positive vesicles transporting key apical determinants. (**a**–**h**) MDCK cells were transfected for 72 h with plasmids encoding either fluorescently tagged Rab35^Q67L^ or Rab35^S22N^ fused to the mitochondrial targeting signal of ActA (-Mito), as indicated. In the merged images, Rab35-Mito proteins are displayed in red, and PODXL, GFP-Crumbs3, GFP-Cdc42 or aPKC in green. (**i**,**j**) MDCK cells were treated with either control or PODXL siRNAs for 72 h, and co-transfected for 72 h with plasmids encoding mCherry-Rab11^WT^ and either GFP-Rab35^Q67L^-Mito or GFP-Rab35^S22N^-Mito, as indicated. (**k**,**l**) MDCK cells were co-transfected for 72 h with plasmids encoding mCherry-Rab35^Q67^-Mito and either GFP-PODXL WT or GFP-PODXL V496A Y500A. In the merged images, Rab35^Q67L^-Mito is displayed in red, and PODXL^WT^ or mutant in green. For **a**–**l** individual channels in grey levels and higher magnification of the regions delimited by a dash line are displayed, as indicated. Scale bars, 10 μm for unzoomed regions and 2 μm for zoomed regions. Arrowheads indicate examples of close-apposition vesicles (green) with mitochondrial Rab35^Q67L^ (red).

Given the physical interaction between Rab35 and the PODXL cytoplasmic tail, as well as the unique localization of Rab35 at the cleavage furrow before PODXL vesicle fusion, these results raised the exciting possibility that Rab35 might act as a direct tether that would capture internalized PODXL vesicles, and thus determine AMIS and lumen location at the cyst centre. To test this tethering model, we experimentally delocalized a GTP-locked mutant of Rab35 to the mitochondrial membrane, by fusing it to the mitochondria-targeting signal of *Listeria monocytogenes* ActA (ref. 28; Rab35^Q67L^-Mito). As expected, this Rab35-chimera localized exclusively to the mitochondrial surface in MDCK cells (Fig. 5a–l). Strikingly, expression of this construct was sufficient to tether vesicles positive for PODXL at the surface of mitochondria, these vesicles appeared as dotty structures closely apposed to the smooth staining of Rab35^Q67L^-Mito (Fig. 5a arrowheads). Importantly, no PODXL vesicles were tethered around mitochondria if the GDP-bound mutant of Rab35 was used (Rab35^S22N^-Mito; Fig. 5b) or when GTP-locked Rab11A^Q70L^-Mito was expressed (Supplementary Fig. 5a). Using live cell imaging, we confirmed that individual PODXL vesicles were tethered around mitochondria by Rab35^Q67L^-Mito, but not by Rab35^S22N^-Mito (Supplementary Movie 4). Using Rab35^Q67L^-Mito to tether PODXL vesicles to mitochondria, we found that these vesicles also contained crucial apical determinants such as GFP-Crumbs3 (Fig. 5c), GFP-Cdc42 (Fig. 5e) and aPKC (Fig. 5g). This was not observed in Rab35^S22N^-Mito-expressing cells (Fig. 5d,f,h). Furthermore, Rab11-postive vesicles were also tethered around mitochondria by Rab35^Q67L^-Mito but not by Rab35^S22N^-Mito (Supplementary Fig. 5b,c), consistent with PODXL and Rab11 being trafficked in the same vesicles^9^,^10^,^14^. As expected for a direct tethering through Rab35 interaction with the PODXL cytoplasmic tail, the tethering of Rab11-positive vesicles crucially depended on the presence of PODXL, since it was lost upon PODXL depletion (Fig. 5i,j). To confirm the importance of the PODXL/Rab35 interaction in vesicle tethering, we analysed cells in which endogenous PODXL had been replaced by either siRNA-resistant GFP-PODXL V496A/Y500A or GFP-PODXL WT. As anticipated, in contrast to vesicles containing GFP-PODXL WT (Fig. 5l), vesicles containing GFP-PODXL V496A/Y500A failed to be tethered around mitochondria by Rab35^Q67L^-Mito (Fig. 5k). Altogether, we conclude that GTP-bound Rab35 tethers intracellular vesicles containing key apical markers (PODXL, aPKC, Cdc42 and Crumbs3) through physical interaction with the cytoplasmic tail of PODXL.

### Delocalizing Rab35 on mitochondria prevents AMIS formation

**Figure 6:**
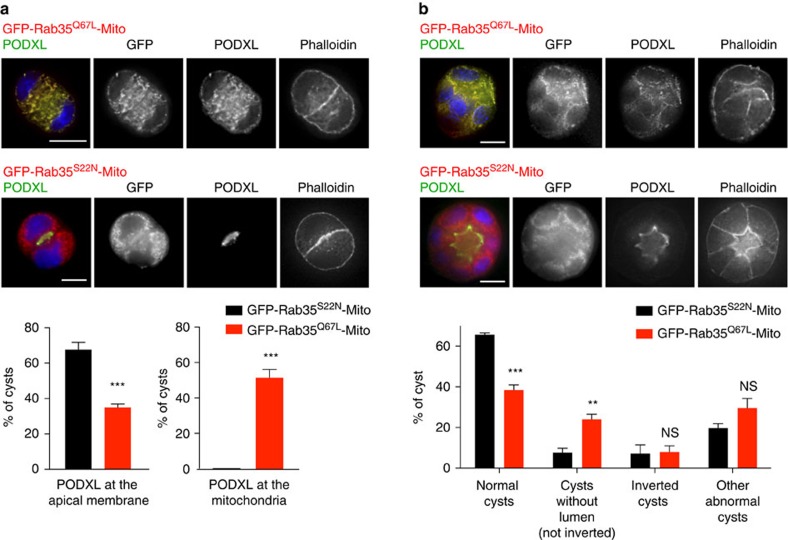
Delocalizing GTP-bound Rab35 on mitochondria prevents AMIS formation and apico-basal polarity establishment. (**a**) MDCK cells transfected with either GFP-Rab35^S22N^-Mito or GFP-Rab35^Q67L^-Mito were seeded into Matrigel for 24 h, cysts were fixed and stained for PODXL and F-actin (phalloidin). Merged images and single channels in grey levels are displayed, as indicated. Scale bar, 10 mm. Graphs: proportion of cysts with PODXL normally localized at the apical membrane (left graph) or with PODXL localized at the mitochondria (right graph) in each condition. Mean±s.d., *N*=3 independent experiments, 100–200 two-cell cysts analysed per condition. Two-way analysis of variance (ANOVA): ***P<0.001. (**b**) MDCK cells were transfected for 24 h with plasmids encoding either GFP-Rab35^Q67L^-Mito or GFP-Rab35^S22N^-Mito and seeded into Matrigel for 48 h. Cysts were then fixed and stained for PODXL (green), F-actin (phalloidin, red) and DNA (DAPI). Merged images and individual channels in grey levels are displayed. Scale bar, 10 mm. Graphs: normal cysts, cysts without lumen, inverted cysts and other abnormal cysts were quantified based on PODXL and Phalloidin staining. Two-way ANOVA: **P<0.01; ***P<0.001; NS, not significant. Mean±s.d., *N*=3 independent experiments, >100 cysts analysed per condition.

If this Rab35-dependent tethering mechanism is important for apical membrane determination, one should expect profound polarity defects when Rab35 is delocalized from the plasma membrane. We thus analysed the consequences of delocalizing Rab35 at mitochondria during cystogenesis. Interestingly, we observed that the proportion of cysts with PODXL correctly localized at the AMIS was strongly reduced upon Rab35^Q67L^-Mito expression, in line with ∼50% of cysts displaying PODXL vesicles at mitochondria (Fig. 6a, 24-h cysts). This was accompanied by the absence of an apical membrane, as shown by a continuous β-catenin staining at the cell–cell interface (Supplementary Fig. 5d,f,h,j, arrows). While Par3 localized to the tight junctions, other apical proteins such as Crumbs3, Cdc42 and aPKC were not correctly localized in the centre of the two-cell-stage cyst in Rab35^Q67L^-Mito-expressing cells (Supplementary Fig. 5d,f,h,j). As expected, apical determinants localized normally at the two-cell stage in cysts expressing Rab35^S22N^-Mito (Supplementary Fig. 5e,g,i,k). As a consequence, expression of Rab35^Q67L^-Mito resulted in the appearance of disorganized cysts without lumen, since PODXL-positive vesicles were tethered around mitochondria throughout cyst development (Fig. 6b, 48-h cysts). In contrast, PODXL was normally enriched at the AMIS after Rab35^S22N^-Mito expression (Fig. 6a), and cysts with a PODXL-positive apical lumen developed normally in this condition (Fig. 6b). Thus, delocalizing GTP-bound Rab35 on mitochondria displaced PODXL-positive vesicles from the plasma membrane, which prevented AMIS formation and apico-basal polarity establishment.

## Discussion

Understanding how apical polarity is established *de novo* and how lumens form in epithelial organs represent crucial biological questions^1^,^2^,^3^,^4^,^5^. In certain organs, such as the acini of exocrine glands, lumens result from the apoptosis of inner glandular cells, through a process called cavitation^1^. In contrast, lumen formation can arise in other tissues through hollowing, for instance, during the development of vascular endothelia and kidneys^1^. In the developing mouse aorta, apical membrane establishes *de novo* from pre-existing cell–cell contacts by targeted exocytosis of apical cargos towards the AMIS^27^. In this system, lumen opening involves PODXL, whose anti-adhesive properties promote cell–cell repulsion. During kidney embryogenesis, the existence of an AMIS has also been described before lumen formation and its elongation into a tubular nephron^29^. MDCK cells form cysts very similar to the embryonic renal vesicle, thus making them an attractive model to study apico-basal establishment and lumen morphogenesis *in vitro*. This led to the key concept that MDCK cysts establish an apical membrane at the two-cell stage through transcytosis of apical proteins, including PODXL, which will later delimit an open lumen^9^,^10^ (Fig. 2b). Interestingly, transcytosis events are temporally and spatially associated with the positioning of the cytokinetic bridge microtubules, which likely promote the delivery of apical recycling endosomes towards the AMIS at the centre of the two-cell cyst^14^. Accordingly, kinesin-2-dependent apical trafficking of the Rab11/FIP5-positive endosomes towards the AMIS during cytokinesis is important for the formation of a single, central apical lumen^12^,^15^. Thus, the formation of the AMIS, which is defined by the tight junction marker ZO-1 (ref. 12), Par3/aPKC and the exocyst subunit Sec8 (ref. 10), must be coordinated with cytokinesis. However, the molecular mechanisms coupling cell division, delivery of apical initiation determinants and lumen formation remained elusive.

**Figure 7:**
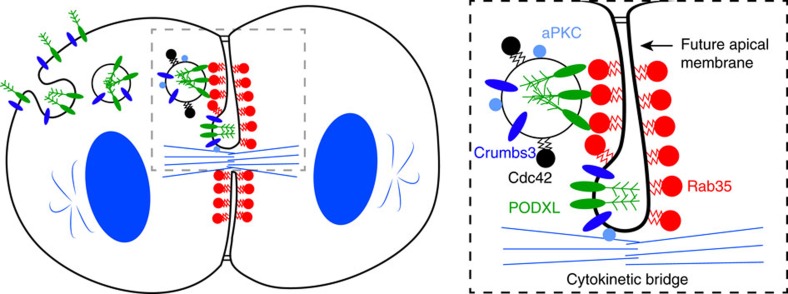
Model for coupling the first cyst cytokinesis and the initiation of the apical lumen at the centre of cysts. Rab35 located at the cell–cell interface surrounding the first intercellular bridge (red) directly tethers internalized PODXL-positive vesicles (green) containing Crumbs3, aPKC and Cdc42 through direct binding to the PODXL cytoplasmic tail. This allows the targeting of vesicles transporting both lumen-promoting factors and key apical determinants to initiate apical polarity at the centre of the cyst-founding cell.

Here we propose that Rab35 acts as a molecular tether that captures PODXL-containing vesicles around the cytokinetic bridge of the cyst-founding cell (Fig. 7). This simple model explains how lumen formation is coupled with the position of the first division apparatus, at the centre of the cyst. Rab35 is indeed localized at the AMIS labelled by ZO-1 around the cytokinetic bridge (Supplementary Fig. 1a). There, we observed co-localization between Rab35 and β-catenin (Supplementary Fig. 1b, arrowheads). This suggests that at least at early stages when PODXL has not yet fused to the AMIS (Supplementary Fig. 1a,b), the AMIS overlaps with adherens junction markers. This observation has been confirmed with co-localization between ZO-1 and β-catenin on the side of the tubulin-positive bridge (Supplementary Fig. 1c). Later, after establishment of the apical membrane (positive for PODXL), there is a clear segregation between adherens junction markers and apical markers (Supplementary Fig. 1d), suggesting remodelling of the junctions. Thus, the AMIS is a dynamic compartment and its molecular definition changes with time. In addition, a pool of Rab35 also localizes at the cell–cell interface not labelled by ZO-1 (Supplementary Fig. 1b). Interestingly, we observed that PODXL vesicles are tethered/fused mainly at the ZO-1-positive AMIS (as expected) but also on its sides (Supplementary Fig. 1e, arrowheads), where Rab35 is also present. This is consistent with the proposed tethering model, and suggests that a pool of POXL vesicles can also interact with β-catenin/Rab35-positive membranes close to the AMIS. This raises the possibility that vesicles could deliver PODXL mainly at the membrane surrounding the bridge (AMIS) but also on its sides, and that apical proteins later coalesce into a single domain while junctions are being remodelled.

Contrary to known tethering machineries (for example, exocyst) that link membranes via assembly of protein sub-complexes found on either membrane, Rab35 tethers vesicles through a physical, direct interaction with the cytoplasmic tail of one of their transmembrane cargo. This thus represents a new mode of tethering, through *trans* interaction between the acceptor compartment and intracellular vesicles. Indeed, Rab GTPases have been shown to be essential regulators of traffic by promoting vesicle formation, targeting and fusion of Rab-positive vesicles with the acceptor compartment^18^,^19^. Here we provide the evidence that Rab GTPases can unexpectedly act as direct and specific receptors for vesicles. Several examples of Rab/transmembrane cargo interactions have been reported (for example, Rab4/CFTR cystic fibrosis chloride channel), but their mechanistic significance remains largely unclear^30^. This novel tethering mode could thus resolve the function of Rab/cargo interactions beyond cyst development. Interestingly, nothing was known about Rab35 in epithelial polarity, except that it regulates branching of polarized seamless tracheal tubes in *Drosophila*^31^. We show here that Rab35 plays a critical role for selectively defining the destination of vesicles that initiate the establishment of polarity. Of note, Rab35 has been involved in the trafficking of a number of cargoes from the endosomes, such as TfR or CI-MPR (refs 16, 17, 32, 33, 34). However, there is no effect of Rab35 depletion on endogenous PODXL or GFP-PODXL internalization from the plasma membrane (Supplementary Fig. 6a–d). Thus, the inversion of polarity observed after Rab35 depletion does not result from the retention of PODXL at the ECM-facing membrane. In addition, depletion of the two known Rab35 effectors in trafficking (OCRL (ref. 32) and MICAL-L1 (refs 33, 35)) did not lead to polarity inversion (Supplementary Fig. 6e–g), as previously shown for OCRL in pure Matrigel cultures^36^. This suggests that Rab35 depletion does not impair PODXL trafficking through early endosomes, as reported for TfR and CI-MPR in HeLa cells^32^, and is in agreement with the presence of transcytosed PODXL in recycling endosome after Rab35 depletion (Fig. 4c and Supplementary Fig. 3a). Altogether, these observations are consistent with a function of Rab35 in targeting PODXL vesicles generated from the Rab11-positive recycling compartment to the future apical membrane (Fig. 7). Of note, the fusion of PODXL vesicles to the AMIS occurs between 1 and 10 h after the metaphase–anaphase transition in control situations, depending on the cysts (Supplementary Fig. 3b). The same cyst-to-cyst variability is observed in Rab35-depleted cysts, until internalized PODXL is fused back to the ECM-facing membrane (Supplementary Fig. 3b). Thus, the process of apical membrane establishment in wild-type cysts is robust but the timing varies.

Our study reveals that PODXL plays a pivotal and broader role in cyst development than previously expected. First, PODXL plays a role in lumen opening, likely by its negatively charged, anti-adhesive extracellular domain^25^,^26^,^27^. Second, our data support the idea that PODXL, directly or more likely indirectly, is necessary for the fusion of the vesicles to the plasma membrane. Indeed, in the absence of PODXL, vesicles containing Crumbs3 do not fuse with the plasma membrane, but rather appear close to the cortex (Fig. 4g), confirming previous reports^25^. Third, PODXL directly participates in the tethering of vesicles containing key determinants for the establishment of the apico-basal polarity to the future apical surface (this study). Accordingly, PODXL-positive vesicles transport other transmembrane apical proteins (for example, Crumbs3) and also a pool of the cytosolic apical determinants such as Cdc42 and aPKC (Fig. 5 and refs 10, 37). While vesicular transport (in that case through the PODXL/Rab35 interaction) is the only way for targeting transmembrane apical proteins, this mechanism also possibly contributes to target a pool of Cdc42/aPKC at the future apical domain. However, other pathways (such as the PtdIns(4,5)*P*_*2*_–Annexin2–Cdc42 pathway^3^) likely function in parallel to deliver cytosolic apical determinants. The importance of PODXL-vesicle fusion at the first cell–cell interface likely explains why Rab35, together with multiple but non-redundant mechanisms of tethering (Rab11/exocyst, Rab11/FIP5/cingulin and Rab27/Slp4-a (refs 7, 10, 11, 12, 38)), is essential for correct initiation of apico-basal polarity in cysts. While Rab35 appears as a major factor promoting the establishment of the apical domain, it is likely the combination of different tethering and fusion factors, together with bridge microtubules and directed molecular motors^12^,^15^, that collectively allow the definition of a robust and properly localized apical membrane.

Complete inversion of cyst polarity has been described in only a few instances^39^,^40^,^41^,^42^,^43^,^44^,^45^. In particular, depletion of β1-integrin interrupts laminin signalling from the extracellular matrix cues and leads to inverted cysts when cultured in pure collagen I matrices^41^. As Rab35 regulates β1-integrin recycling in HeLa cells^46^,^47^, one can wonder whether Rab35 depletion could additionally perturb polarity by preventing β1-integrin trafficking to the cell surface. This is unlikely since Rab35 depletion, on the opposite, increases recycling of β1-integrin to the plasma membrane^46^. In addition, PODXL is not known to be involved in β1-integrin trafficking and replacing endogenous PODXL by a PODXL mutant that cannot interact with Rab35 is sufficient to induce polarity inversion, even without depleting Rab35 (Fig. 4f,h). Finally, further work revealed that β1-integrin depletion does not lead to complete inversion of polarity but rather to impaired polarity reorientation characterized by collective front–rear polarization and motility^25^, when MDCK cells are cultured in Matrigel (our conditions).

Rab35 depletion and overexpression of the Rab35GAP EPI64B experiments revealed that both Rab35 and its level of activation are important for correct establishment of apico-basal polarity and lumen localization (Fig. 3c and Supplementary Fig. 2d,e). This is fully consistent with the observation that PODXL interacts specifically with active, GTP-bound Rab35 (Fig. 1a–c). Interestingly, the Rab35 GAP EPI64B can directly bind to NHERF1/EBP50 (ref. 48), which itself interacts with Ezrin and PODXL at the ECM-facing membrane^25^. It is possible that the former interaction contributes to maintain low levels of GTP-bound Rab35 at the ECM-facing membrane. Conversely, since Rab35 is directly recruited by PtdIns(4,5)*P*_*2*_ (ref. 49), it is likely that high levels of PtdIns(4,5)*P*_*2*_ at the cleavage furrow, as well as the conversion of PtdIns(3,4,5)*P*_*3*_ into PtdIns(4,5)*P*_*2*_ via PTEN at the apical plasma membrane^3^ promote Rab35 localization at this site.

Finally, Rab35 and PODXL have been found to be differentially expressed in several cancers: Rab35 is downregulated in several tumours, including renal carcinomas^46^,^50^, whereas PODXL overexpression has been identified as a potent marker for highly aggressive and metastatic cancers with poor prognosis^51^,^52^. Since perturbed polarity is associated with tumour progression, the finding that Rab35 directly interacts with PODXL and that its inactivation perturbs both PODXL targeting and polarity may explain the emerging role of Rab35 and PODXL in tumorigenesis^46^,^50^,^51^,^52^,^53^.

*Note added in proof:* The Fukuda laboratory identified through comprehensive RNAi screens the Rab GTPases mediating podocalyxin transcytosis and showed that different sets of Rabs, including Rab35, coordinate its transport during cell polarization in 2D and 3D MDCK cells. For more details, see: Mrozowska P. and Fukuda M. Regulation of podocalyxin trafficking by Rab small GTPases in 2D and 3D epithelial cell cultures. *The Journal in Cell Biology*, in press (2016).

## Methods

### Antibodies and plasmids

The following antibodies were used for immunocytochemistry experiments: mouse anti-PODXL (DSHB Hybridoma 3F2/D8 deposited by G.K. Ojakian); human anti-acetylated-tubulin C3B9-hFc (Institut Curie, Paris, France); goat anti-β-catenin (sc-31,000; Santa Cruz Biotechnology); rabbit anti-ZO-1 (61-7300, Invitrogen); rabbit anti-Par3 (07-330, Millipore); mouse anti-E-cadherin (610181; BD Transduction Laboratories); rabbit anti-aPKCζ (C-20, Santa Cruz); mouse anti-integrin β1 (4B4, Coulter Clone); mouse anti-CTR433 (a kind gift from M. Bornens, Institut Curie, Paris, France); and recombinant hVHH anti-GFP-hFc (Institut Curie, Paris, France). Fluorescently labelled secondary antibodies were purchased from Jackson Laboratories. Phalloidin 647 was purchased from Sigma-Aldrich. Mouse anti-β-tubulin (Clone Tub 2.1, Sigma-Aldrich), mouse anti-6xHis (Clone HIS-1, Sigma-Aldrich), mouse anti-GFP (11814460001; Roche), mouse anti-Flag (M2, Sigma-Aldrich), rabbit anti-Rab35 (described in ref. 16) and secondary horseradish-peroxidase-coupled antibodies (Jackson Laboratories) were used for western blot experiments.

The following plasmids were used for the yeast two-hybrid experiments presented in Fig. 1a: pGAD-rabbit PODXL cytoplasmic tail (aa 476–551); pGAD-rabbit PODXL V496A Y500A cytoplasmic tail (aa 476–551); pLex-human wild-type Rab35 K173 (aa 1–173); pLex-human Rab35^Q67L^ K173 (aa 1–173); pLex-human Rab35^S22N^ K173 (aa 1–173); pLex-human Rab11^Q70L^ Y173 (aa 1–173); pLex-human Rab6A^Q72L^ (aa 1–173); pLex-human Rab8A^Q67L^ (aa 1–183); and pLex-human Rab27A^Q78L^ (aa 1–201). Plasmids used for immunofluorescence were the following: pmCherry-human Rab35^WT^ (siRNA-resistant)^17^; pmCherry-human Rab35^S22N^ (siRNA-resistant)^17^; pEGFP-rabbit wild-type PODXL (a kind gift from K. Simons); GFP-human Crumbs3 (a kind gift from A. Le Bivic); peGFP-human Cdc42 (a kind gift from S. Etienne-Manneville); pmCherry Rab11 (a kind gift from B. Goud); peGFP canine Rab11 (a kind gift from Z. Lenkei); pEGFP-EPI64B^WT^; pEGFP-EPI64B^R409A^; and pmCherry-tubulin. The plasmids for the mitochondria-targeting experiments pGFP-Rab35^Q67L^-Mito, pmCherry-Rab35^Q67L^-Mito, pGFP-Rab35^S22N^-Mito, pmCherry-Rab35^S22N^-Mito, pGFP canine Rab11A^Q70L^-Mito and pmCherry canine Rab11A^Q70L^-Mito were constructed by PCR as followed: cDNA encoding Rab proteins lacking their last cysteins Rab35^S22N^ (aa 1–200), Rab35^Q67L^ (aa 1–200) and Rab11^Q70L^ (aa 1–212) were fused at their COOH terminus to DNA encoding the mitochondrial targeting sequence ‘LILAMLAIGVFSLGAFIKIIQLRKNN' of *L. monocytogenes* ActA. All constructs were Rab35 shRNA-resistant. All mutations were obtained by Quickchange (Invitrogen).

### RNA interference

The following siRNAs were used: canine Rab35, 5′- GCTCACGAAGAACAGTAAA -3′ (Sigma-Aldrich); canine PODXL, 5′- CACTGGAAGTGATGGAGACCT -3′ (ref. 26; Sigma-Aldrich); canine Cep55, 5′- AGCAAGAAATCAAATAACA -3′ (Smartpool Sigma-Aldrich); canine MICAL-L1, 5′- GAGAGAAGGTGCTGATGCA -3′ (ref. 54; Sigma-Aldrich); canine OCRL, 5′- GGTTCCCTGCCATTTTTCA -3′ (ref. 36; Sigma-Aldrich); and control luciferase, 5′- CGUACGCGGAAUACUUCGA -3′ (Sigma-Aldrich). The following shRNAs were used: control shRNA, 3′- GTCTCCACGCGCAGTACATTT -5′; and Rab35 shRNA, 3′- CTGGTCCTCCGAGCAAAGAAA -5′ (Amsbio). Lentiviral particles of pLenti-H1-shRNA-Rsv-IRES-Pyromycin and pLenti-H1-shRNA-Rsv-IRES-GFP-Pyromycin were provided by Amsbio. The shRNA expression is driven by a tetracycline inducible cytomegalovirus promoter. Rab35 siRNAs were transfected using Amaxa 2 days before seeding cells into Matrigel. Cep55 and PODXL siRNAs were transfected 24 and 48 h before seeding cells into Matrigel.

### Yeast two-hybrid screen and experiments

A yeast two-hybrid screen with human Rab35^Q67L^ (aa 1–194) fused to LexA as a bait was carried out by Hybrigenics SA (Paris, France), using a human placenta complementary DNAGal4-activating domain (GAD)-fusion library^17^. Specificity experiments (Fig. 1a) were performed by co-transforming the *Saccharomyces cerevisae* reporter strain L40 with either pGAD-rabbit PODXL (aa 476–551) or pGAD alone together with either pLex-human wild-type Rab35 (aa 1–173), pLex-human Rab35^Q67L^ (aa 1–173), pLex-human Rab35^S22N^ (aa 1–173), pLex-human Rab11^Q70L^ (aa 1–173), pLex-human Rab6A^Q72L^ (aa 1–173), pLex-human Rab8A^Q67L^ (aa 1–183) or pLex-human Rab27A^Q78L^ (aa 1–201). The later plasmids encode for Rab proteins without their COOH-terminal hypervariable regions. Transformed yeast colonies were selected on DOB agarose plates without tryptophan and leucine. Colonies were picked and grown on DOB agar plates with histidine (to select co-transformants) and without histidine (to detect interactions).

### Cell cultures and transfections

MDCK II cells (ATCC) were grown in alpha-MEM medium supplemented with 10% fetal bovine serum, 100 U ml^−1^ pernicillin/streptomycin and 200 mM glutamine. Cells were transfected using Amaxa Kit L (Lonza) following the manufacturer's instructions.

### Lentiviral transductions and stable cell lines

A stable cell line expressing mCherry-Rab35 was obtained by transfection using AMAXA, followed by selection with 100 ng μl^−1^ Genetecin (G418) for 2 weeks and FACS sorting for mCherry-positive cells. Stable cell lines expressing control shRNA IRES GFP, Rab35 shRNA IRES GFP, control shRNA or Rab35 shRNA are obtained by lentiviral transduction (10 multiplicity of infection, 10^7^ particles per ml) in growth medium and 5 μg ml^−1^ Polybrene, following selection with 1 μg ml^−1^ Pyromycin 72 h after transduction. Cells expressing control shRNA IRES GFP or Rab35 shRNA IRES GFP were sorted by FACS for low, medium and high levels of GFP fluorescence intensities.

### Two-dimensional cell culture and immunofluorescence

MDCK cells grown on coverslips were fixed with 4% paraformaldehyde (PFA) for 10 min at room temperature, quenched with 50 mM NH_4_Cl for 20 min, permeabilized in 0.1% Triton X-100 for 3 min and incubated in blocking buffer (0.2% BSA, 0.05% saponin and PBS) for 20 min. In Fig. 5a–l and Supplementary Fig. 5a–c, cells were stained with a primary antibody mouse anti-PODXL (1:1,000) and rabbit anti-aPKCζ (1:1,000) in blocking buffer for 1 h, washed with PBS, incubated with a secondary antibody (1:1,000) in blocking buffer for 1 h, washed, stained with DAPI (4,6-diamidino-2-phenylindole) and mounted with Mowiol.

### 3D cultures in Matrigel and immunofluorescence

MDCK cells were resuspended in culture medium containing 0.3 mg ml^−1^ phenol red-free Matrigel (Corning) and seeded into Matrigel-coated eight-well chamber slides (Milicell EZ slide eight-well glass; Millipore) at a concentration of 6,000 single cells per well. Chamber slides were coated with 10 μl pure Matrigel per well and incubated at 37 °C for 2 min before cell seeding. The cells were incubated for the indicated period of time before fixation with 4% PFA for 30 min at room temperature. Cells were then treated with 50 mM NH_4_Cl for 20 min, permeabilized with 0,5% Triton X-100 for 15 min and incubated with a primary antibody in PBS 0.3% saponin for 2 h at room temperature followed by 1 h incubation with a secondary antibody (1:1,000) or labelled Phalloidin (1:4,000) in PBS 0.3% saponin. Nuclei were stained with DAPI for 5 min, chambers were removed and the slides were mounted with Mowiol. The following concentration of primary antibodies were used: mouse anti-PODXL (1:1,000); human anti-acetylated-tubulin (1:200); goat anti-β-catenin (1:200); rabbit anti-ZO-1 (1:200); rabbit anti-Par3 (1:200); mouse anti-E-cadherin (1:200); rabbit anti-aPKCζ (1:1,000); mouse anti-integrin β1 (1:200); and mouse anti-CTR433 (1:50).

### Immunofluorescence microscopy and deconvolution

Images were acquired with an inverted Ti E Nikon microscope, using a × 100 1.4 NA (numerical aperture) PL-APO objective lens or a × 60 1.4 NA PL-APO VC objective lens and MetaMorph software (MDS) driving a CCD (charge-coupled device) camera (Photometrics Coolsnap HQ)^55^,^56^. *Z*-stacks were acquired with a distance of 130 μm. The 16-bit images were deconvolved using Huygens Professional software (SVI) to reduce off-plane background fluorescence (2–20 iterations, signal/noise 40). Images were then converted into 8-bit images using ImageJ software (NIH). Images in Fig. 4g, Supplementary Figs 1, 2d, 5d-k, 6f were acquired using an inverted Eclipse Ti E Nikon microscope equipped with a CSU-X1 spinning disk confocal scanning unit (MDS), driving a EMCCD Camera (Evolve 512 Delta, Photometrics) Images were acquired with a × 100 1.4 NA PL-APO objective lens and MetaMorph software (MDS).

### Time-lapse microscopy

Two-dimensional cultures (Supplementary Movie 4 and Supplementary Fig. 4a): For time-lapse phase-contrast microscopy, transient or stably transfected MDCK cells were plated on 35-mm glass dishes (MatTek) and put in an open chamber (Life Imaging) equilibrated in 5% CO_2_ and maintained at 37 °C. Time-lapse sequences were recorded every 5 min for 48 h (Supplementary Fig. 2a) or every second (Supplementary Movie 4) using a Nikon Eclipse Ti inverted microscope with a × 20 0.45 NA Plan FluorELWD objective (Supplementary Fig. 4a) or a × 100 1.4 NA PL-APO objective (Supplementary Movie 4) controlled by Metamorph 6.1 software (Universal Imaging). This microscope was equipped with a cooled CCD camera (HQ2; Roper Scientific). In Supplementary Fig. 4a, cytokinetic abscission time was quantified in mCherry-tubulin-transfected cells by time-lapse microscopy starting from furrow ingression until the cut of the intracellular bridge. In all, 100 cells per condition were analysed. Supplementary Movie 4 was deconvolved using Huygens Professional software SVI (2 iterations, signal/noise 10).

### 3D cultures for time-lapse microscopy

Transient or stably transfected MDCK cells were resuspended in culture medium containing 0.3 mg ml^−1^ phenol red-free Matrigel (Corning) and seeded into coated glass bottom 24-well plates (MatTek) at a concentration of 10,000 single cells per well. The glass was coated with poly-L-lysine-γ-polyethyleneglycol (SuSoS) to create an anti-adhesive surface, followed by coating with 5 μl pure Matrigel before cell seeding. Time-lapse sequences were recorded with a Nikon Eclipse Ti inverted microscope at 5 min for 24 h, × 100 1.4 NA PL-APO objective. Image sequences were deconvolved using Hyugens Professional software (SVI) as described above and assembled with ImageJ software (Supplementary Movie 1–3).

### Antibody internalization assay

MDCK cells stably expressing control shRNA or Rab35 shRNA (Supplementary Fig. 6a,b) were plated into six-well plates (500,000 cells per well), washed with cold medium, incubated on ice for 10 min and then incubated with anti-PODXL antibodies 1:400 for 30 min on ice. MDCK cells were washed twice with cold medium; warm medium was added and cells were shifted at 37 °C for various time points. Cells were then shifted back on ice and remaining surface-bound antibodies were removed by treatment with 1 mg ml^−1^ Pronase (Sigma-Aldrich) for 30 s on ice. The cells were fixed with 4% PFA for 10 min at room temperature, quenched with 50 mM NH_4_Cl for 20 min, permeabilized in 0.1% Triton X-100 for 3 min, incubated in blocking buffer (0.2% BSA, 0.05% saponin and PBS) for 20 min and stained with secondary anti-mouse PE antibodies (1:200) before FACS analysis. FACS analysis was carried using MoFlo Astrios, FACS machine (Beckman Coulter) and FlowJo software. For Supplementary Fig. 6c,d: MDCK cells stably expressing Rab35 shRNA were transfected with GFP-PODXL WT for 24 h; MDCK cells stably expressing control shRNA were depleted of endogenous PODXL using siRNAs for 3 days and transfected with either GFP-PODXL WT or GFP-PODXL^V496A/Y500A^ for 24 h. The antibody internalization and FACS analysis was performed as described above, but using anti-GFP primary antibodies (recombinant hVHH anti-GFP-hFc; 1:400) and anti-human PE secondary antibodies.

### Co-immunoprecipitation assays

MDCK cells were transfected with either 3xFlag-tagged Rab35^WT^, Rab35^S22N^, Rab35^Q67L^, Rab11A^Q70L^, Rab6A^Q72L^, Rab8A^Q67L^ or Rab27A^Q78L^ for 24 h using Amaxa. Cells were lysed in 25 mM Tris (pH 7.5), 150 mM NaCl, 10 mM MgCl_2_ and 0.1% Triton X-100 (lysis buffer). Post-nuclear supernatants (20 min at 20,000*g*) were incubated with anti-Flag M2 affinity gel (Sigma-Aldrich) for 2 h, washed with lysis buffer, resuspended into 1 × Laemmli buffer and boiled at 95 °C for 5 min. The amount of co-immunoprecipitated PODXL in each condition was probed by western blot analysis using anti-Flag antibodies (1:2,000).

### Bacterial expression and recombinant protein purification

6xHis–Rab11A^WT^ full-length, 6xHis–Rab6A^WT^ full-length, 6xHis–Rab35^WT^ full-length, GST–PODXL WT (aa 476–551), GST–PODXL V4496A Y500A (aa 476–551) or glutathione *S*-transferase (GST) alone were expressed in the BL21 pLysS strain of *Escherichia coli* after induction with 1 mM isopropyl-β-D-thiogalactopyranoside at 37 °C for 3 h. Cells were lysed with PBS containing 1 mg ml^−1^ lysozyme, 1 mM dithiothreitol and protease inhibitor (Roche) by sonication on ice. The GST fusion proteins were affinity-purified using glutathione Sepharose 4B (GE Healthcare) and eluted with 20 mM HEPES at pH 7.5, 150 mM NaCl and 20 mM reduced glutathione. 6xHis-fused proteins were affinity-purified using Ni-NTA Magnetic Agarose Beads (Qiagen) and were eluted in 50 mM Tris (pH 8), 150 mM NaCl, 2 mM MgCl_2_ and 250 mM imidazole.

### GST-pull down and western blot experiments

6xHis–Rab35^WT^, 6xHis–Rab11A^WT^ and 6xHis–Rab6A^WT^ were exchanged with either 1 mM GDP or 200 μM GTPγS in 25 mM Tris (pH 7.5), 100 mM NaCl, 10 mM EDTA, 5 mM MgCl_2_ and 1 mM dithiothreitol for 1 h at 37 °C. Nucleotides were then stabilized with 20 mM MgCl_2_. GST–PODXL WT (aa 476–551), GST–PODXL V496A Y500A (aa 476–551) or GST alone were loaded onto glutathione Sepharose 4B beads (Pharmacia) in 25 mM Tris (pH 7.5), 1 mM MgCl_2_ and 0.2% BSA for 1 h at 4 °C. Beads were then incubated with exchanged 6xHis-Rab proteins in 25 mM Tris (pH 7.5), 50 mM NaCl, 10 mM MgCl_2_, 0.1% Triton X-100 and 0.2% BSA. Beads were washed, resuspended into 1 × Laemmli buffer and boiled at 95 °C for 5 min. Pulled-down 6xHis–Rab proteins were detected by western blot using anti-6xHis antibodies (1:5,000) and GST-tagged proteins loaded on beads were detected by Ponceau red staining. Full scans of all western blots are displayed in Supplementary Figs 7 and 8.

### Statistical analysis

MDCK cysts were scored into categories and displayed as mean proportions±s.d. from three independent experiments. Significance was calculated using a two-way analysis of variance with a Tukey *post hoc* test. For abscission times, a non-parametric Kolmogorov–Smirnov test was used. In all statistical tests *P*>0.05 was considered as not significant.

## Additional information

**How to cite this article:** Klinkert, K. *et al*. Rab35 GTPase couples cell division with initiation of epithelial apico-basal polarity and lumen opening. *Nat. Commun.* 7:11166 doi: 10.1038/ncomms11166 (2016).

## Supplementary Material

Supplementary Figures Supplementary Figures 1-8

Supplementary Movie 1 MDCK cells stably expressing mCherry-Rab35^WT^ were transiently transfected with GFP-PODXL and seeded into Matrigel. Images were acquired every 5 min for 24 h. Bar: 5 mm. Time in [hour:min].

Supplementary Movie 2 Stable Rab35 shRNA MDCK cells were transiently transfected with GFP-PODXL and image sequences were acquired as for Movie 1. Time in [hour:min].

Supplementary Movie 3 MDCK cells were co-transfected with PODXL siRNA and with plasmids encoding mCherry-Rab35^WT^ and siRNA resistant GFP-PODXL V496A/Y500A. Image sequences were acquired as for Movie 1. Bar: 5 mm. Time in [hour:min].

Supplementary Movie 4 MDCK cells were co-transfected with plasmids encoding GFP-PODXL WT and either mCherry-Rab35^Q67L^-Mito or mCherry-Rab35^S22N^-Mito as indicated and image sequences were acquired every second for 1 min. Bar: 5 mm. Time in [min:sec].
