# Supplementary material for: Rab35 GTPase couples cell division with initiation of epithelial apico-basal polarity and lumen opening
Source: Nat Commun. 2016 Apr 4;7:11166. doi: 10.1038/ncomms11166 (PMC4822036; doi:10.1038/ncomms11166)
Supplement: Supplementary Figures — 1-8 [file ncomms11166-s1.pdf]

SUPPLEMENTARY INFORMATION

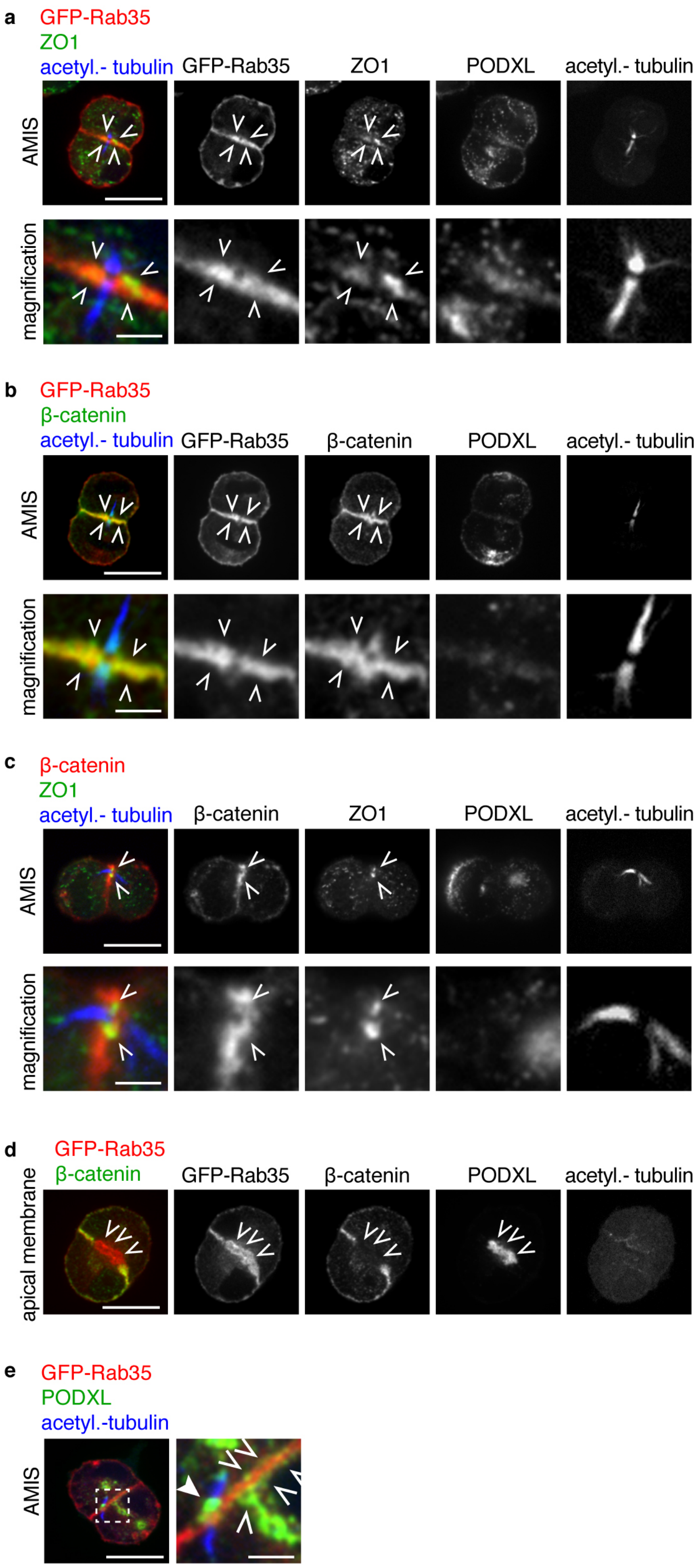

Supplementary Figure 1

Supplementary Figure 1: Rab35 is present at the AMIS

MDCK cells stably expressing GFP-Rab35^{WT} were cultured in Matrigel for 16h and stained for PODXL, acetylated tubulin and ZO1 **(a)** or b-catenin **(b,d)**. Arrowheads indicate the localization of the AMIS (a-b) or the apical membrane (d). Bar: 10 μm (zoom: 2 μm) **(c)** MDCK cells were cultured in Matrigel for 16h and stained for PODXL, acetylated tubulin, ZO1 and b-catenin. **(e)** MDCK cells stably expressing GFP-Rab35^{WT} (displayed in red) were cultured in Matrigel for 16h and stained for PODXL (green) and acetylated tubulin (blue). Magnification shows the accumulation of PODXL-vesicles around the Rab35 positive membrane. Bar: 10 μm (zoom: 2 μm)

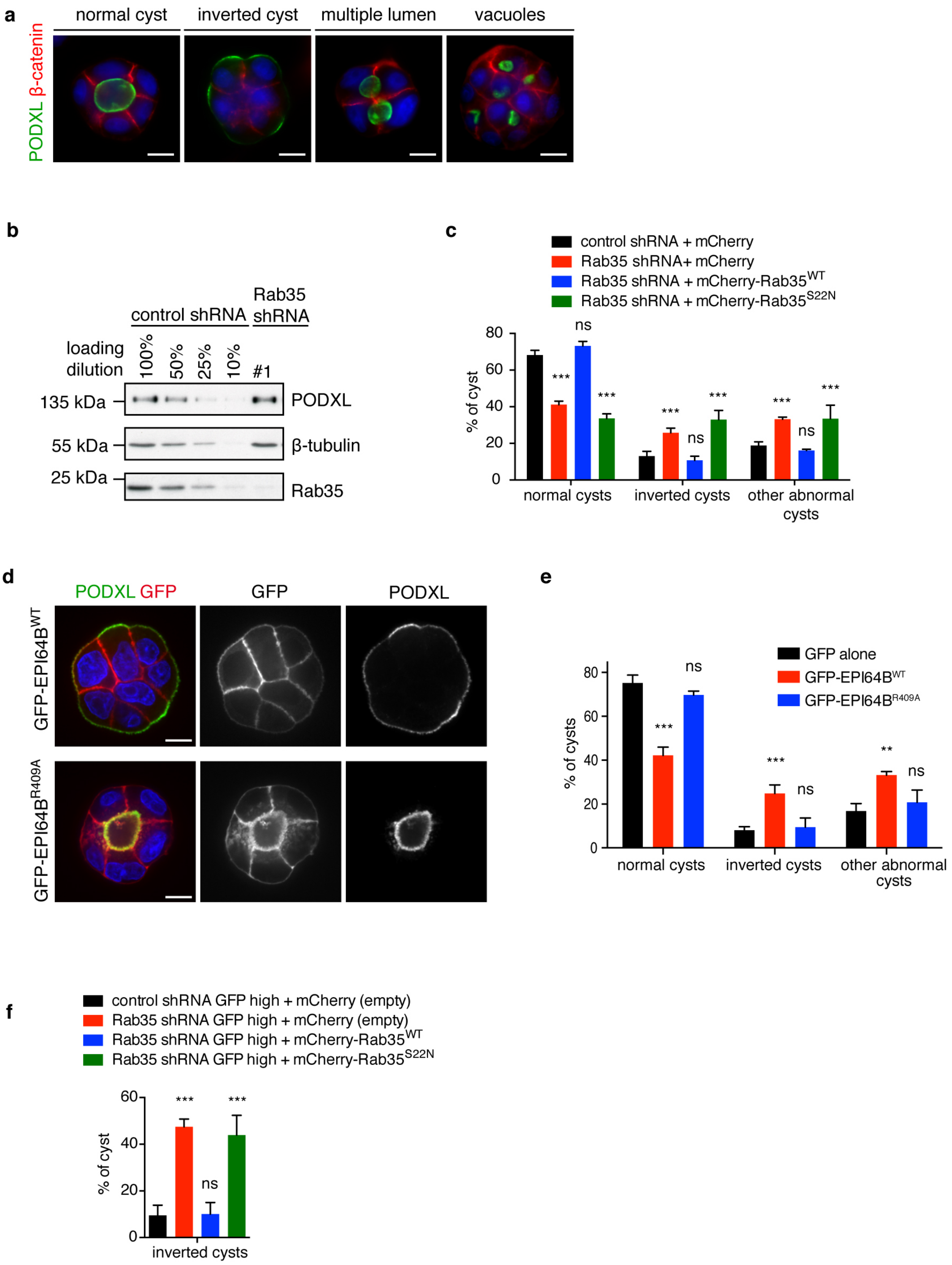

Supplementary Figure 2

Supplementary Figure 2: Rab35 depletion by either siRNAs or shRNAs leads to abnormal cyst development.

(a) MDCK cells were treated with Rab35 siRNAs for 48 h and seeded into Matrigel for 48 h. Cysts were fixed and stained for PODXL (green), β-catenin (red) and DNA (DAPI, blue). Examples of normal cysts, inverted cysts and other abnormal cysts (multiple lumen shared by neighbouring cells or with intracellular vacuoles) upon Rab35 depletion. Bar: 10 μm. **(b)** Western blot of MDCK cell lysates that stably express shRNAs targeting *Rab35* mRNA, using antibodies detecting PODXL, β-tubulin and Rab35, as indicated. 100% or dilutions of the lysate from control shRNA were loaded for comparison (lysate from Rab35 depletion corresponds to 100%). Rab35 depletion reduced by >90% of endogenous Rab35 levels. **(c)** MDCK cells that stably expressed either shRNAs targeting luciferase (control) or *Rab35* mRNAs were transfected with plasmids encoding mCherry alone, siRNA-resistant mCherry-$Rab35^{WT}$ or siRNA-resistant mCherry-$Rab35^{S22N}$, as indicated. 48 h after seeding in Matrigel, the proportion of normal cysts, inverted cysts or other abnormal cysts based on PODXL staining was quantified. Mean ± SD, N= 3 independent experiments, 300-600 cysts analysed per condition. Two-way ANOVA: p<0.001(***), ns: not significant. **(d)** MDCK cells were transfected with either GFP-$EPI64B^{WT}$, GFP-$EPI64B^{R409A}$ or GFP alone and seeded into Matrigel for 48h. Cysts were fixed and stained for PODXL. Merged images and single channels in grey levels are displayed, as indicated. Bar: 10 μm. **(e)** Proportion of normal cysts with a single lumen, inverted cysts and other abnormal cysts in each condition. Mean ± SD, N= 3 independent experiments, >500 cysts analysed per condition. Two-way ANOVA: p<0.001(***),p<0.01(**). **(f)** MDCK cells that stably expressed either control shRNA IRES GFP or Rab35 shRNA IRES GFP were sorted for high levels of fluorescence (GFP high). Cells were then transfected with plasmids encoding mCherry alone, shRNA-resistant mCherry- $Rab35^{WT}$ or shRNA-resistant mCherry-$Rab35^{S22N}$, as indicated, and seeded into Matrigel for 48 h. The proportion of inverted cysts based on PODXL staining was quantified in each condition. Mean ± SD, N= 3 independent experiments, 100-600 cysts analysed per condition. Two-way ANOVA: p<0.001(***), ns: not significant.

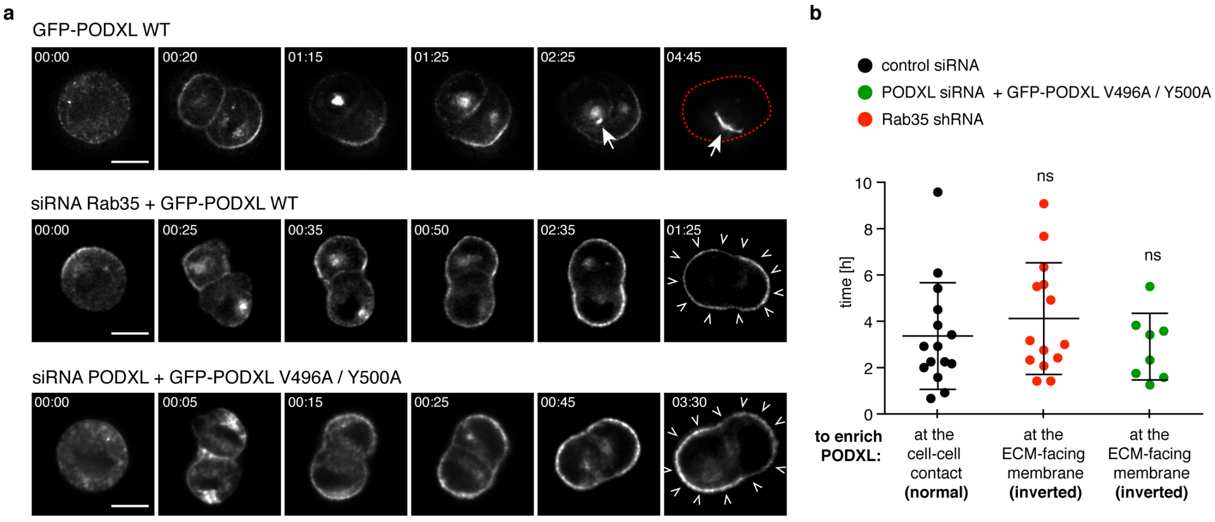

Supplementary Figure 3

Supplementary Figure 3: Additional time-lpase examples of control and Rab35-depleted cysts, and time to form the first apical membrane.

(a) Snapshots of time-lapse microscopy (as in Fig. 4) from control MDCK cells expressing GFP-PODXL^{WT} seeded into Matrigel (top row), Rab35-depleted cells expressing GFP-PODXL^{WT} (middle row) and PODXL-depleted cells expressing GFP-PODXL$^{V496A\ Y500A}$ (bottom row). Arrows indicate the apical membrane in control cysts and arrowheads the inverted apical membrane in Rab35 depleted or PODXL mutant expressing cells . Bar: 10 μm. Time stamps: [hour:min] using mitotic entry as origin. **(b)** Quantification of the time to establish an apical membrane from mitosis onset until PODXL enrichment at the cell-cell contact (control cysts) or PODXL enrichment at the ECM-facing membrane (Rab35 depleted cysts or GFP-PODXL mutant expressing cysts).

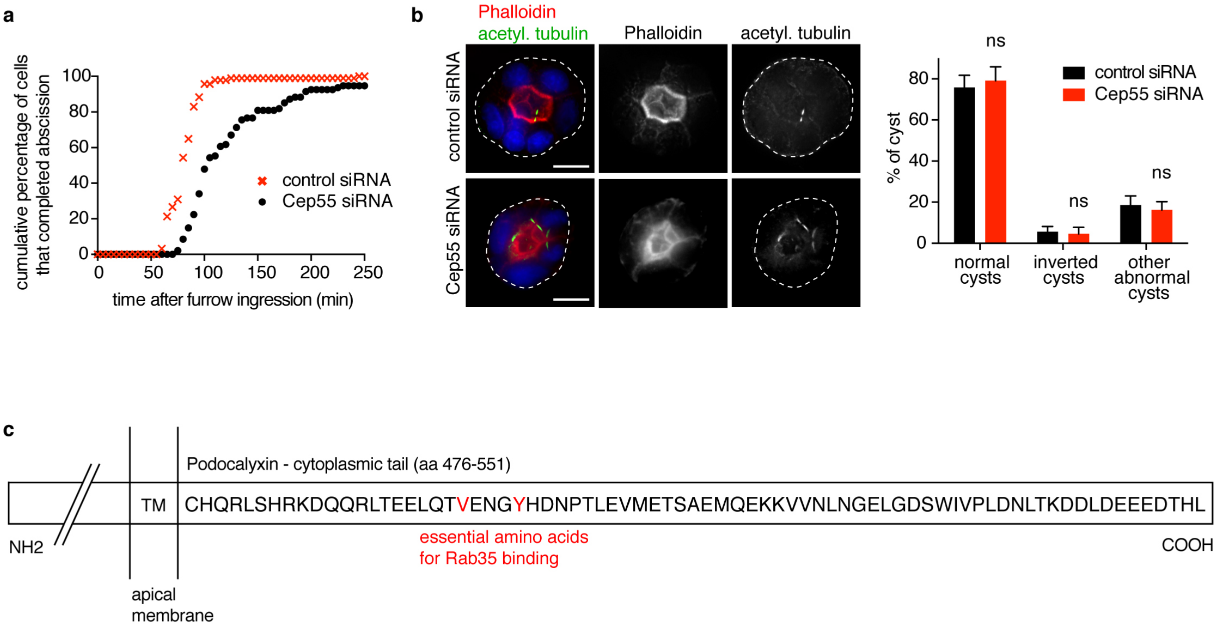

Supplementary Figure 4

Supplementary Figure 4: Defects in cytokinesis abscission does not perturb cyst development.

(a) MDCK cells were treated with either control (red) or Cep55 (black) siRNAs for 72 h and recorded by phase-contrast time-lapse microscopy for 48 h. Abscission time counted from furrow ingression to actual abscission (x-axis) are presented as cumulative percentage (y-axis). Note the strong delay in abscission observed after Cep55 depletion. **(b)** MDCK cells were treated with either control or Rab35 siRNAs for 48 h and seeded into Matrigel for 48 h. Left panels: Cysts were fixed and stained with phalloidin to detect apical F-actin (red), anti-acetylated-tubulin antibodies to visualize cytokinetic bridges (green) and DAPI (blue). Projections of z-stacks are presented. Single channel for acetylated-tubulin is also displayed in grey levels. Note the accumulation of cytokinetic bridges upon Cep55 depletion, indicating delayed cytokinesis in 3D cysts. Bar: 10 μm. Right panel: Proportion of normal and abnormal cysts after control or Cep55 depletion, based on PODXL staining. Mean ± SD, N= 3 independent experiments, 300-500 cysts analysed per condition. Two-way ANOVA: ns: not significant. **(c)** Carboxy-terminal sequence of PODXL cytoplasmic tail (aa 476-551). The position of Valine 496 and Tyrosine 500 essential for Rab35 binding are indicated in red.

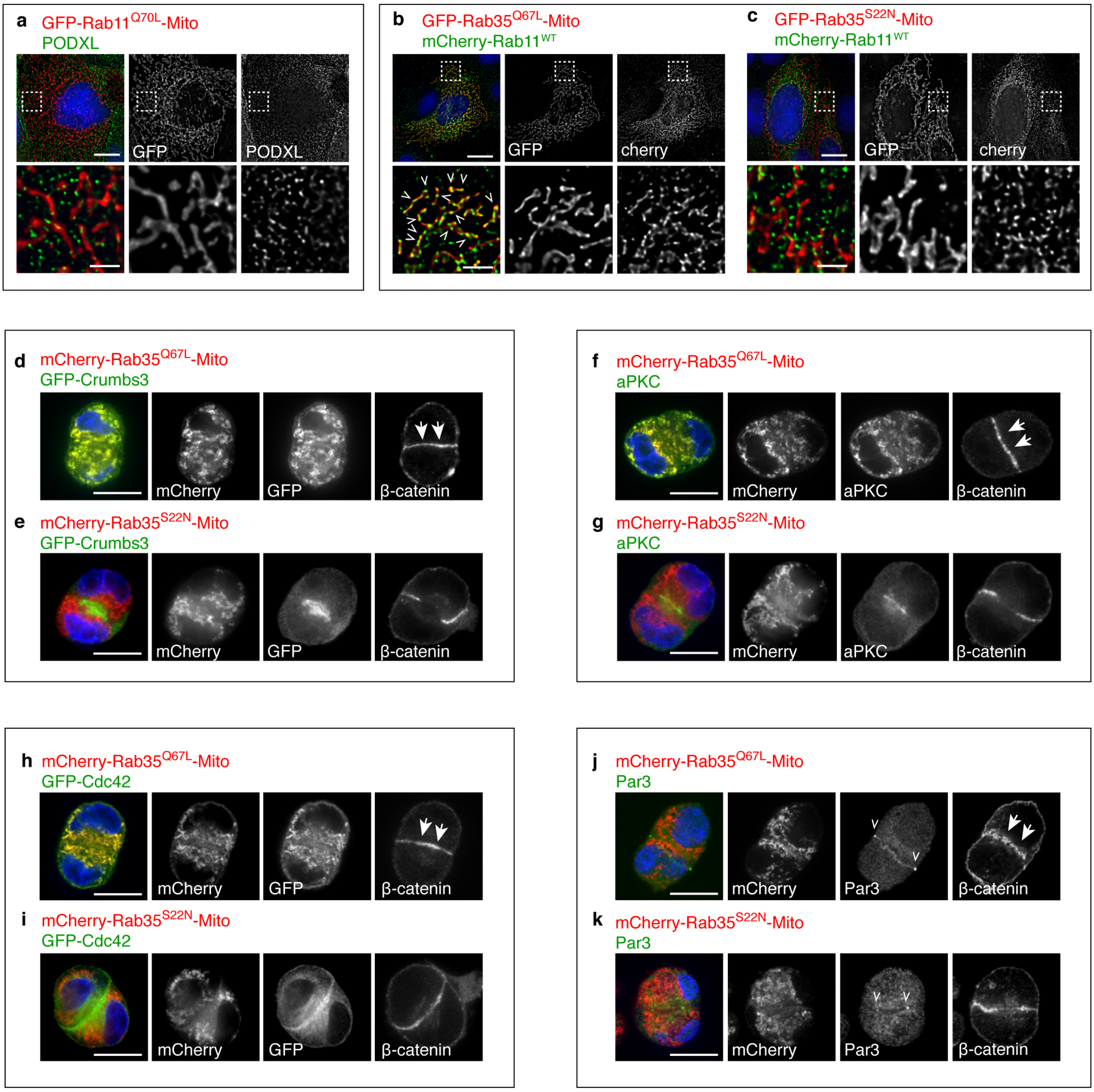

Supplementary Figure 5

Supplementary Figure 5: Rab35^{Q67L}-Mito tethers vesicles containing PODXL and Rab11 around mitochondria.

(a) MDCK cells were transfected for 72 h with plasmids encoding GFP-Rab11^{Q70L} fused to the mitochondrial targeting signal of ActA (-Mito), fixed and stained for PODXL. **(b-c)** MDCK cells were co-transfected for 72 h with plasmids encoding mCherry-Rab11^{WT} and either GFP-Rab35^{Q67L}-Mito **(b)** or GFP-Rab35^{S22N}-Mito **(c)**, fixed and stained with DAPI. **(a-c):** merged images, individual channels in grey levels and higher magnification of the regions delimited by a dash line are displayed, as indicated. Bar: 10 μm for unzoomed regions and 2 μm for zoomed regions. Arrowheads indicate examples of close apposition vesicles (green) with mitochondrial Rab35^{Q67L} (red). **(d-k)** MDCK cells were co-transfected with plasmids encoding either GFP-Rab35^{Q67L}-Mito (d, f, h, j) or GFP-Rab35^{S22N}-Mito (e, g, i, k), together with GFP-Crumbs3 (d-e) or GFP-Cdc42 (h-i). Cells were seeded into Matrigel for 24h, fixed and stained with β-catenin (d-e, h-i), β-catenin and aPKC (f-g) or β-catenin and Par3 (j-k) as indicated. Arrows indicate the β-catenin marked baso-lateral membrane. Bar: 10 μm

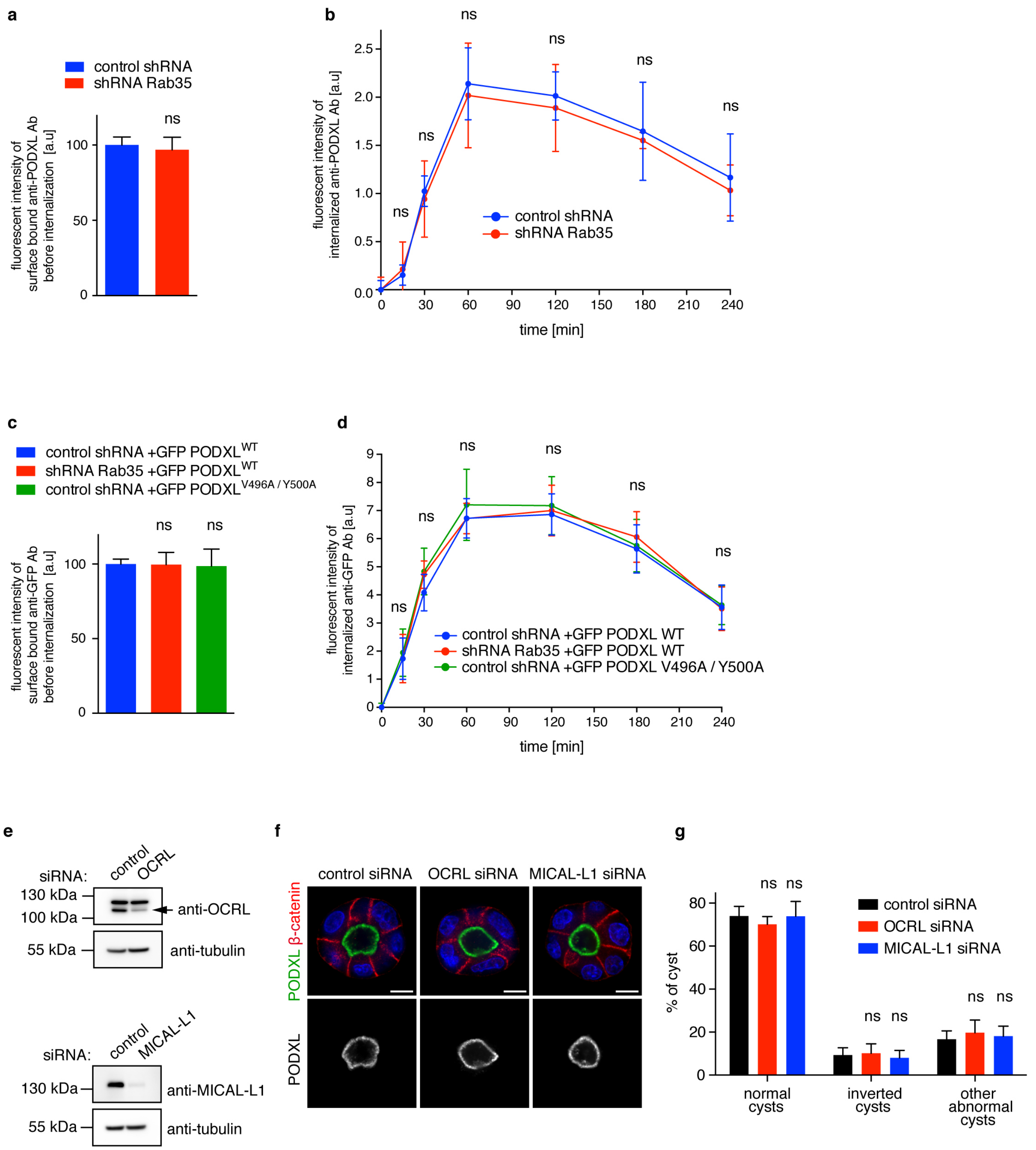

Supplementary Figure 6

Supplementary Figure 6: Rab35 depletion does not affect PODXL endocytosis.

(a-b) MDCK cells stably expressing control shRNA or Rab35 shRNA were incubated with anti-PODXL antibodies at 4°C and then shifted to 37°C to allow antibody internalization for various time points as indicated. Remaining surface bound antibodies were removed by pronase treatment (except for baseline control cells were fixed, permeabilized and stained with secondary fluorescent antibodies before being analysed by FACS. The graph in (a) presents the fluorescent intensity of surface bound anti-PODXL antibody before internalization. **(c-d)** Endogenous PODXL was depleted with siRNAs and replaced by either GFP-PODXL^{WT} (blue line) or GFP-PODXL$^{V496A\ Y500A}$ (green line) in MDCK cells stably expressing control shRNA. The red line shows MDCK cells stably expressing Rab35 shRNA that were transfected with GFP-PODXL^{WT} (red line). Internalization of exogenous PODXL was analyzed as in (a-b), by using anti-GFP antibodies. All graphs present triplicates of representative experiments. Mean ± SD. In b and d, values are normalized to baseline (surface bound antibodies before internalization) and substracted from background fluorescent at t0. **(e)** Western Blot of MDCK cells transfected with either OCRL siRNA, MICAL-L1 siRNA or control siRNA for 3 days. **(f)** MDCK cysts transfected with either OCRL siRNAs, MICAL-L1 siRNAs or control siRNAs were seeded into Matrigel for 48h and stained for PODXL (green), β-catenin (red), DAPI (blue). Bar: 10 µm **(g)** Proportion of normal cysts, inverted cysts and other abnormal cysts of control cysts, OCRL-depleted cysts and MICAL-L1-depleted cysts after 48h in Matrigel. Mean ± SD, N= 3 independent experiments, >300 cysts analysed per condition. Two-way ANOVA: ns: p>0.05.

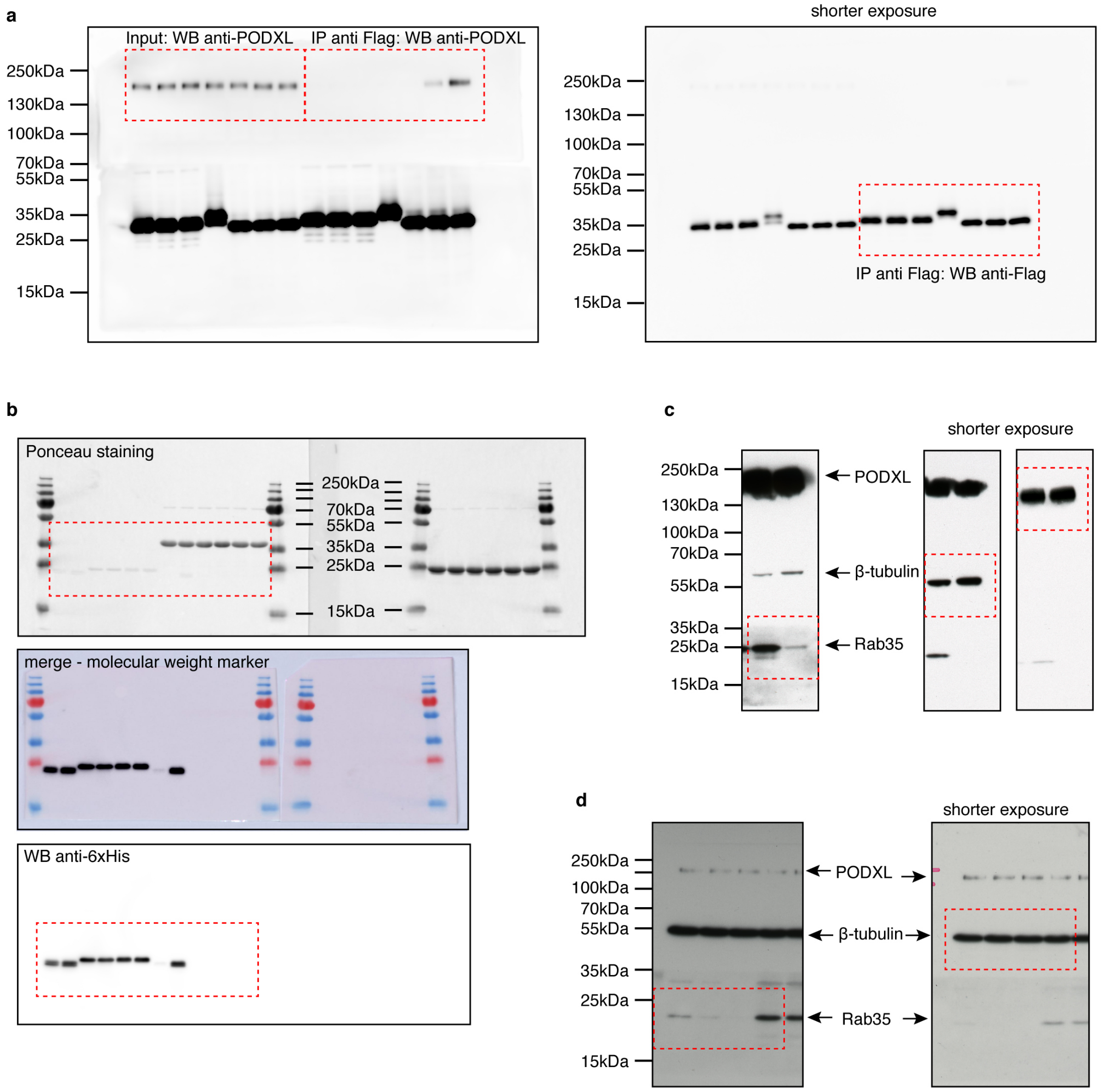

Supplementary Figure 7

Supplementary Figure 7: uncropped images for presented Western Blots (I)

(a) corresponds to Fig. 1c; **(b)** corresponds to Fig. 1b; **(c)** corresponds to Fig. 3a; **(d)** corresponds to Fig. 3d. Red squares indicate cropped regions as displayed in the Figures.

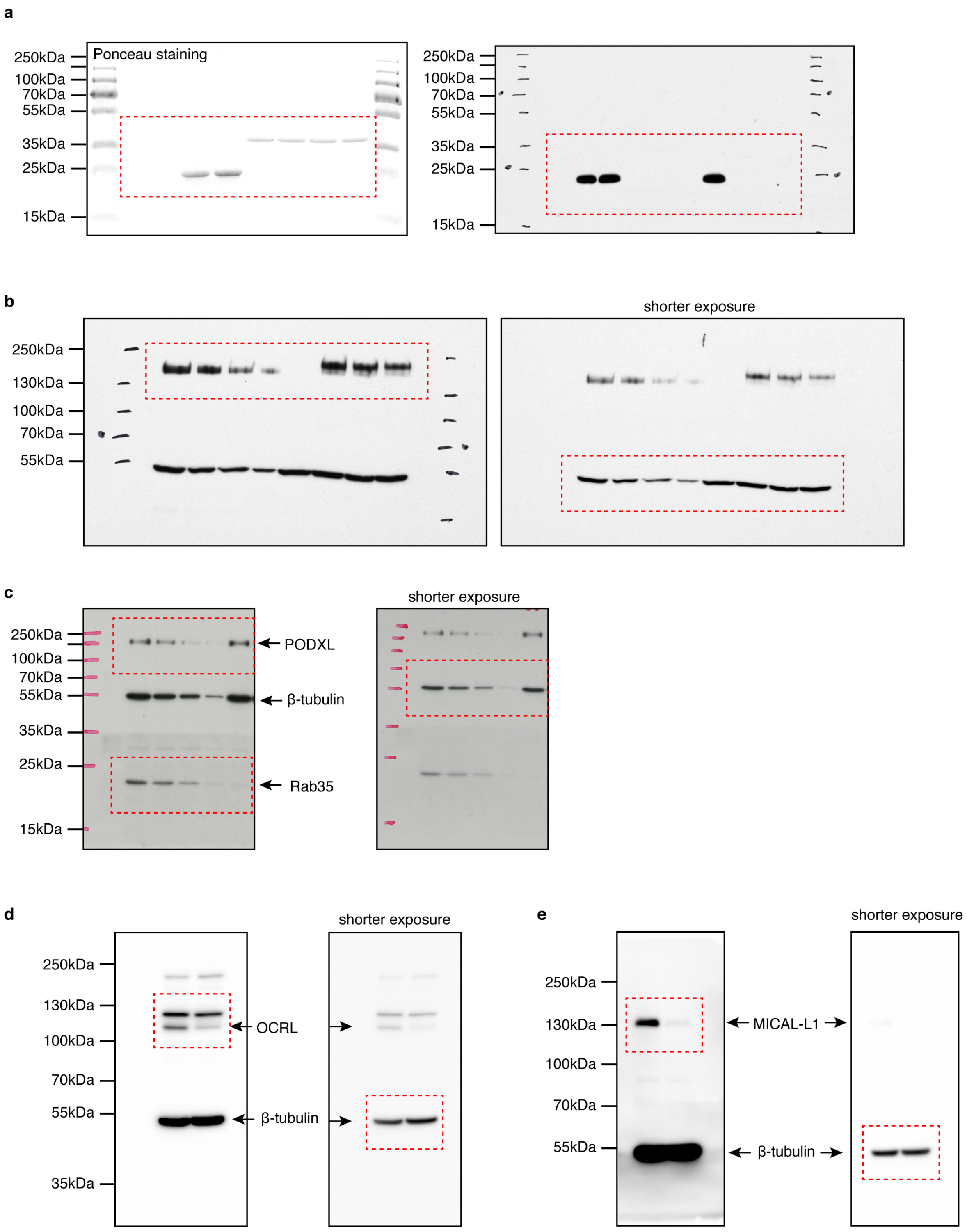

Supplementary Figure 8

Supplementary Figure 8: uncropped images for presented Western Blots (II)

(a) corresponds to Fig. 4d; **(b)** corresponds to Fig. 4e; **(c)** corresponds to Supplementary Fig. 2b; **(d-e)** corresponds to Supplementary Fig. 6e. Red squares indicate cropped regions as displayed in the Figures.
